# Nanoalbumin–prodrug conjugates prepared via a thiolation‐and‐conjugation method improve cancer chemotherapy and immune checkpoint blockade therapy by promoting CD8 ^+^ T‐cell infiltration

**DOI:** 10.1002/btm2.10377

**Published:** 2022-07-30

**Authors:** Long Chen, Nuo Xu, Pan Wang, Haichuan Zhu, Zijian Zhang, Zhanqun Yang, Wenyuan Zhang, Hongyan Guo, Jian Lin

**Affiliations:** ^1^ Department of Pharmacy Peking University Third Hospital, College of Chemistry and Molecular Engineering, Peking University Beijing China; ^2^ Department of Obstetrics and Gynecology Peking University Third Hospital Beijing China; ^3^ Institute of Biology and Medicine, College of Life and Health Sciences, Wuhan University of Science and Technology Wuhan China

**Keywords:** CD8^+^ T cells, immune checkpoint blockade, NanoAlb‐proDOX, protein–drug conjugates, thiolation‐and‐conjugation

## Abstract

Protein–drug conjugates are emerging tools to combat cancers. Here, we adopted an indirect thiolation‐and‐conjugation method as a general strategy to prepare protein–drug conjugates. We found for the first time that this method led to the formation of nanometric conjugates, probably due to the formation of intermolecular disulfide bonds, which facilitated enhanced uptake by cancer cells. As a proof‐of‐concept application in cancer therapy, a nanometric albumin–doxorubicin prodrug conjugate (NanoAlb‐proDOX) was prepared. The nanometric size promoted its uptake by cancer cells, and the prodrug characteristic defined its selective cytotoxicity toward cancer cells in vitro and reduced side effects in vivo. In multiple tumor xenograft models, nanometric NanoAlb‐proDOX showed superior antitumor activity and synergy with immune checkpoint blockade, probably due to the synergistically enhanced tumor CD8^+^ T‐cell infiltration and activation. Hence, the thiolation‐and‐conjugation strategy may serve as a generally applicable method for preparing drug conjugates, and the proof‐of‐concept nanometric albumin–doxorubicin conjugate may be a good choice for antitumor therapy with the ability to co‐stimulate the efficacy of immune checkpoint blockade.

## 1 INTRODUCTION

Cancer remains one of the major threats to human life and is the second most common cause of death worldwide each year.^1^ Alongside surgery, chemotherapy and immune checkpoint blockade immunotherapy are two of the main weapons against cancer.^2^, ^3^, ^4^, ^5^ Although extensive breakthroughs have extensively made over the decades,^2^, ^6^ all of these strategies suffer from drawbacks.^7^, ^8^ The adverse side effects, low response rates, and insufficient efficacy greatly hinder their applications.^9^, ^10^, ^11^, ^12^ A rising strategy is the combination of chemotherapy and immune checkpoint blockade, which may synergistically elevate the therapeutic efficacy.^13^ However, the strategy still suffers from adverse side effects induced by chemotherapy.

One emerging strategy to reduce the side effects and to enhance the therapeutic efficacy of chemotherapy is to conjugate small‐molecule chemotherapy drugs to functional proteins to yield protein–drug conjugates,^14^, ^15^ such as antibody–drug conjugates (ADCs).^16^, ^17^, ^18^ Protein–drug conjugates or antibody‐conjugates usually consist of three components: a protein (antibody), a cytotoxic payload (drug), and a chemical linker that connects the two components.^19^ Thus, to produce protein–drug conjugates, specific conjugation methods are essential.^20^ Currently, many conjugation strategies are available. Among them, site‐specific labeling methods, due to their homogeneous production of protein–drug conjugates, have attracted increasing interest.^21^, ^22^ However, the necessity of genetic engineering of the proteins hinders their application to a certain extent.^23^ In contrast, random labeling of reactive amino acid residues, such as lysine or cysteine, with the advantage that no genetic manipulations are needed, is more widely applied.^20^, ^24^ ADCs with linker and payloads directly conjugated to either lysine residues or cysteine residues have been approved.^24^

In addition to direct conjugation of drug payloads to protein, indirect conjugation methods involve a first step of functionalization of the protein of interest with chemical molecules and a second step of conjugation on the functionalized groups. These methods are also widely used because some linker‐payloads are not suitable for direct one‐step conjugation to protein.^25^ A typical example of these indirect conjugation approaches is the thiolation‐and‐conjugation method enabled by the application of the bifunctional crosslinking molecule *N*‐succinimidyl‐3‐(2‐pyridyldithio) propionate (SPDP).^25^, ^26^ The protein of interest is first modified with sulfhydryl groups, and further conjugation of the protein with linker‐payload is accomplished using maleimide‐thiol‐based chemistry. Although the SPDP‐based method has been reported for the production of protein–protein conjugates, its application and versatility as a method to generate protein–drug conjugates for cancer therapy have not been extensively studied. Whether this conjugation method interferes with the properties of the proteins,^27^, ^28^ especially in terms of the tumor targeting ability and internalization ability by cancer cells for application in cancer therapy,^29^ should be well studied. Generally, a conjugation method that either improves the tumor targeting or enhances the uptake of the protein by cancer cells would be greatly preferred.^30^

**FIGURE 1:**
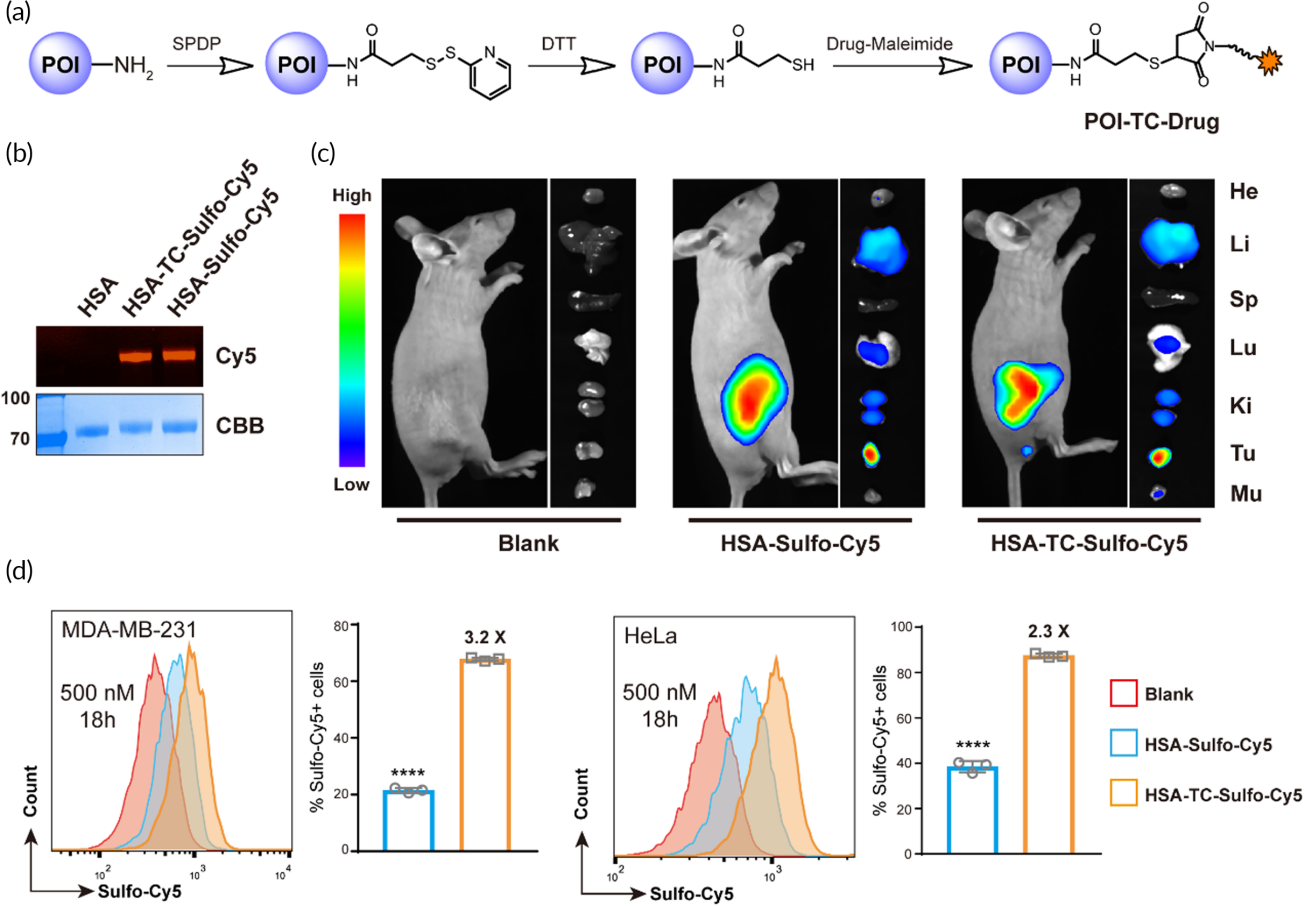
Thiolation‐and‐conjugation do not alter the tumor targeting of the protein of interest but enhance cancer cellular uptake. (a) Schematic illustration of the synthesis of protein–drug conjugates via the thiolation‐and‐conjugation approach. POI, protein of interest. (b) SDS–PAGE analysis of albumin‐sulfo‐cy5 conjugate (HSA‐TC‐Sulfo‐Cy5). HSA‐Sulfo‐Cy5 was prepared through direct conjugation of Sulfo‐Cy5 to lysine residues on HSA. In gel Cy5 fluorescence indicated that HSA was successfully labeled with Sulfo‐Cy5 using two different methods. CBB, Commassie brilliant blue staining; Cy5, Sulfo‐Cy5 fluorescence. (c) In vivo and ex vivo imaging of the tumor targeting of HSA‐TC‐Sulfo‐Cy5. HSA‐Sulfo‐Cy5 or HSA‐TC‐Sulfo‐Cy5 was intravenously injected into mice bearing MDA‐MB‐231 subcutaneous tumors, and fluorescence imaging was performed 24 h post‐injection using a living animal imaging system. No obvious differences in tumor accumulation were observed between HSA‐Sulfo‐Cy5 or HSA‐TC‐Sulfo‐Cy5. (d) Enhanced cellular uptake of HSA‐TC‐Sulfo‐Cy5 by cancer cell lines. Two cancer cells were incubated with the indicated concentrations of protein–dye conjugates for 18 h, and flow cytometry analysis showed that the uptake of HSA‐TC‐Sulfo‐Cy5 was higher than that of HSA‐Sulfo‐Cy5 in both MDA‐MB‐231 and HeLa cells. The marked concentration represents the concentration of Sulfo‐Cy5 added to the cell culture. The percentages of Sulfo‐Cy5‐positive cells were gated, and fold changes between the two conjugates are marked. Data are presented as the mean ± SEM. *n* = 3 technical replicates. ****p < 0.0001

Herein, we applied this SPDP‐based, indirect thiolation‐and‐conjugation method to construct protein–drug conjugates (Figure 1a) and proved its versatility in cancer therapy. Although this approach has been previously reported, we revealed that during the synthesis procedures, the conjugates self‐assembled to form nanometric particles. The nanometric protein–drug conjugates preserved good tumor targeting ability and showed enhanced uptake by cancer cells in vitro. As proof‐of‐concept application in cancer therapy, the nanometric protein–drug conjugate strategy was combined with an acid‐sensitive prodrug strategy to produce an albumin and doxorubicin conjugate: NanoAlb‐proDOX. This nanoconjugate showed preferential cytotoxicity to cancer cells over noncancer cells in vitro and reduced side effects in vivo. In multiple mouse xenograft models, NanoAlb‐proDOX also showed superior antitumor activity over doxorubicin. In a combination therapy model, NanoAlb‐proDOX synergistically elevated the efficacy of immune checkpoint blockade with enhanced infiltration of cytotoxic CD8^+^ T cells into the tumor microenvironment. Overall, the results showed that the thiolation‐and‐conjugation approach can serve as a general method for constructing protein–drug conjugates that self‐assemble into nanoparticles and that the proof‐of‐concept nanoalbumin–doxorubicin conjugate is a possible choice for cancer therapy.

## 2 RESULTS

### 2.1 Preparation of protein–drug conjugates via a thiolation‐and‐conjugation method

To prepare protein–drug conjugates, a thiolation‐and‐conjugation approach was adopted (Figure 1a). As illustrated, the protein of interest was first modified with SPDP on lysine residues and selectively reduced with dithiothreitol (DTT) to first install free sulfhydryl groups. The reduction was performed under slightly acidic conditions that left the native intramolecular disulfide bonds unaffected (see detailed procedure in Section 5.2). Then, sulfhydrylated protein was conjugated with a drug of interest bearing a maleimide warhead. As a proof of concept, human serum albumin (HSA) and the fluorophore Sulfo‐Cyanine 5 (Sulfo‐Cy5) conjugate (HSA‐TC‐Sulfo‐Cy5) were prepared and characterized with sodium dodecyl sulfate‐polyacrylamide gel electrophoresis (SDS–PAGE, Figure 1b). HSA‐Sulfo‐Cy5 with direct Cy5 conjugation was also prepared for comparison. In gel Cy5 fluorescence indicated that HSA was modified by Sulfo‐Cy5 with both methods (Figure 1b).

The effects of the thiolation‐and‐conjugation approach on the tumor targeting ability of HSA were first validated by comparison with HSA‐Sulfo‐Cy5. Breast cancer mouse xenograft models were constructed and utilized. As shown in Figure 1c, both HSA‐Sulfo‐Cy5 and HSA‐TC‐Sulfo‐Cy5 showed good accumulation in tumors, showing that the thiolation‐and‐conjugation method did not affect the tumor targeting ability of the protein of interest and indicating that the thiolation‐and‐conjugation may be a feasible approach to prepare protein conjugates. HSA can be internalized by different cancer cells (Figure S1), which facilitates its application in cancer therapy. Therefore, we further tested whether the thiolation‐and‐conjugation approach affects protein uptake by cancer cells. A flow cytometry assay was performed to quantify the internalization of both HSA‐Sulfo‐Cy5 and HSA‐TC‐Sulfo‐Cy5 by two cancer cell lines. The time‐ and concentration‐dependent uptake of the two HSA conjugates was validated. As shown in Figure 1d and Figure S2, the uptake of HSA‐TC‐Sulfo‐Cy5 exceeded that of HSA‐Sulfo‐Cy5 (2.2‐ to 5.4‐fold, calculated from the percentages of Sulfo‐Cy5‐positive cells) at different concentrations (200 and 500 nM) and time points (3 and 18 h) in both cell lines (MDA‐MB‐231 and HeLa), indicating that the conjugate prepared by the thiolation‐and‐conjugation approach may exhibit improved internalization by cancer cells, which is beneficial to tumor therapy. As another example, we also prepared albumin conjugates with nonsulfonated cyanine 5 (Cy5) using the two methods (Figure S3A). In a similar uptake assay, HSA‐TC‐Cy5 showed enhanced uptake in HeLa cells compared with that of HSA‐Cy5 after different incubation times (1.6‐ to 1.9‐fold, calculated from the mean fluorescence intensity, Figure S3B,C). To prove that the enhanced cellular uptake was universal to other proteins, we further prepared another conjugate (RBD‐TC‐Sulfo‐Cy5) using the recombinant receptor binding domain (RBD) of SARS‐CoV‐2 due to fast accessibility to this protein (Figure S4). Flow cytometry analysis indicated that uptake of RBD‐TC‐Sulfo‐Cy5 by HeLa cancer cells was also enhanced (2.3‐ to 24.6‐fold in mean fluorescence intensity) compared to that of directly conjugated RBD‐Sulfo‐Cy5 (Figure S4), indicating that the enhanced cellular uptake of the conjugate prepared via thiolation‐and‐conjugation may be universal. Albumin is reported to enter cancer cells through the gp60 receptor, and the RBD of SARS‐CoV‐2 enters cells through the ACE2 receptor; therefore, we speculated that the enhanced cellular uptake may be independent of their receptors. Overall, the above data showed that the thiolation‐and‐conjugation approach can be a universal strategy to generate protein–drug (small molecule) conjugates with the advantage of increasing uptake by cancer cells.

### 2.2 Synthesis and characterization of the NanoAlb‐proDOX conjugate

**FIGURE 2:**
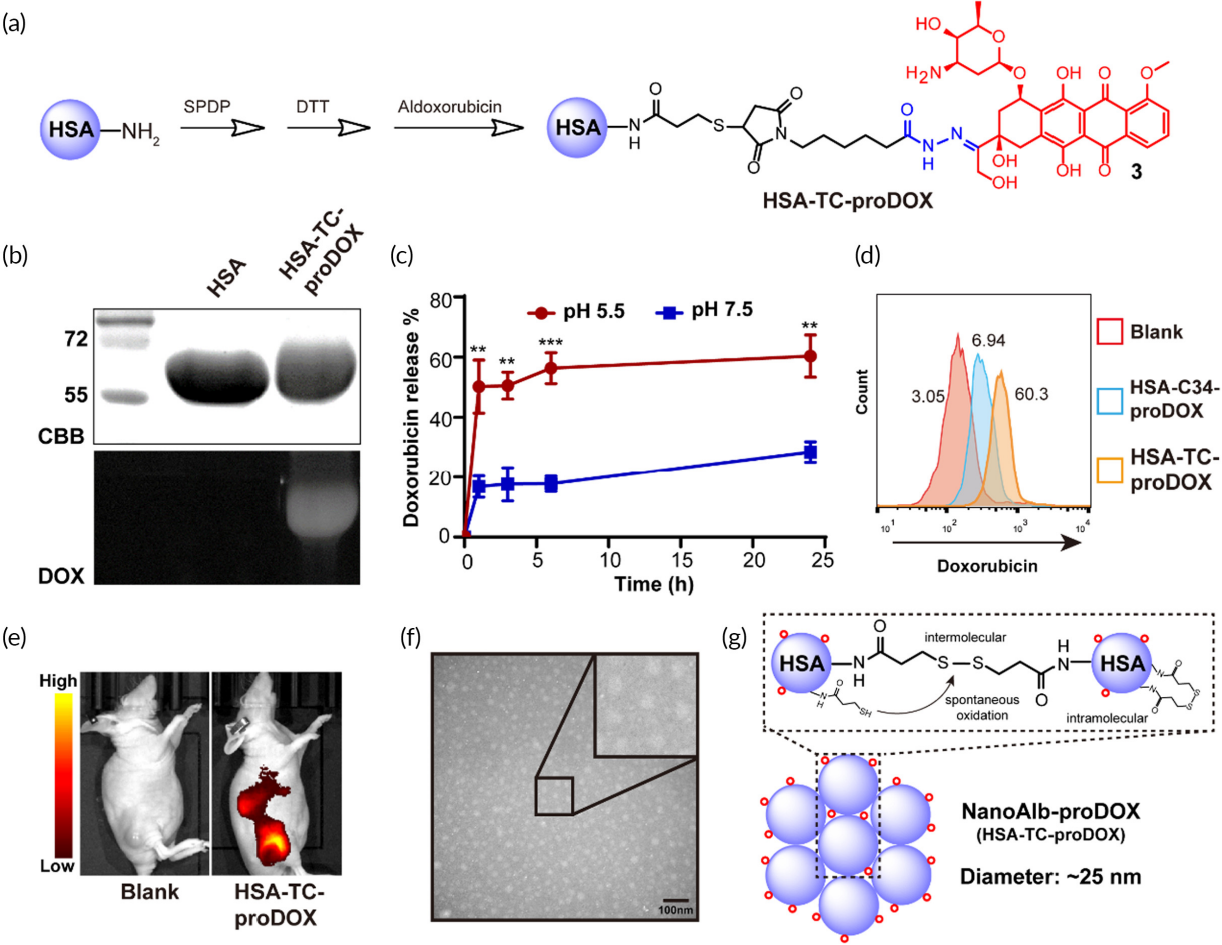
Synthesis and characterization of NanoAlb‐proDOX. (a) Schematic illustration of the synthesis of HSA‐TC‐proDOX. (b) SDS–PAGE analysis of HSA and HSA‐TC‐proDOX. In gel doxorubicin fluorescence of HSA‐TC‐proDOX indicated that HSA was successfully labeled with doxorubicin. CBB, Commassie brilliant blue staining; DOX, doxorubicin fluorescence. (c) pH‐dependent doxorubicin release from HSA‐TC‐proDOX. pH 7.5 and 4.5 were selected to simulate the environment of blood circulation or the tumor microenvironment, respectively. (d) Flow cytometry plots showing enhanced uptake of HSA‐TC‐proDOX by HeLa cancer cells. Conjugate with one micromolar doxorubicin was incubated with HeLa cells for 24 h and analyzed by flow cytometry. The percentages of positive cells are marked. (e) Accumulation of HSA‐TC‐proDOX in tumors. Cy5‐labeled HSA‐TC‐proDOX was intravenously injected into mice bearing MDA‐MB‐231 subcutaneous tumors, and fluorescence imaging of live animals was performed 24 h post‐injection. Accumulation of HSA‐TC‐proDOX in the tumor region was observed. (f) Transmission electron microscopy (TEM) image of HSA‐TC‐proDOX. Scale bar: 100 nm. (g) Proposed mechanism of the nanoparticle formation of HSA‐TC‐proDOX. The nanoparticle was speculated to assemble via the spontaneous oxidation of the excess sulfhydryl groups to form intermolecular disulfide bonds. Intracellular disulfide bonds and residual sulfhydryl groups may also exist. Red circles represent the proDOX molecules. HSA‐TC‐proDOX is hereafter named NanoAlb‐proDOX. Data are presented as the mean ± SEM. *n* = 3 technical replicates. **p < 0.01, ***p < 0.001

Encouraged by the data shown above, we next decided to prepare a real protein–‐drug conjugate and validate its application in cancer therapy. HSA, due to its biocompatibility, was selected as the protein of interest. In addition, a doxorubicin prodrug molecule was utilized to further reduce the side effects.^31^ Therefore, HSA was first introduced with sulfhydryl groups as described above (Figure 2a). Then, a doxorubicin prodrug bearing an acid‐sensitive hydrazone linker, which enables tumor microenvironment‐specific or lysosome‐specific active doxorubicin, and a maleimide warhead were conjugated to sulfhydrylated HSA to generate the HSA‐TC‐proDOX conjugate. The resulting conjugate was first characterized with SDS–PAGE to confirm the modification with doxorubicin, and in gel doxorubicin fluorescence proved the presence of doxorubicin molecules on HSA (Figure 2b). Doxorubicin was fluorescent with a maximum absorption at approximately 498 nm (Figure S5), so the extent of doxorubicin modification was determined by plotting a standard curve of doxorubicin fluorescence and fitting the fluorescence of HSA‐TC‐proDOX to it (Figure S6). A representative dataset is summarized in Table S1. The drug (doxorubicin)‐to‐protein (albumin) ratio was determined to be approximately 3.0. The pH‐dependent and time‐course release of active doxorubicin from NanoAlb‐proDOX, resulting from the acid‐sensitive hydrazone linker, was also measured. Aqueous solutions with pH values of 7.5 or 5.5 were selected to simulate either the pH during circulation or the pH in the tumor microenvironment (or in the lysosome), respectively. As shown in Figure 2c, the conjugate was relatively stable at pH 7.5 (less than 40% doxorubicin released from the protein conjugate in 24 h), while it was quickly triggered to release active doxorubicin at pH 5.5 (approximately 50% doxorubicin released from the protein conjugate within 2 h). These results indicated that the conjugate preferentially releases the drug in the acidic tumor microenvironment and may thus reduce unexpected drug release during circulation, helping to minimize side effects.

As mentioned earlier, the HSA‐TC‐Sulfo‐Cy5 conjugate prepared via the thiolation‐and‐conjugation approach not only did not alter the tumor targeting ability of HSA but also enhanced the uptake of HSA by cancer cells. Similar properties were also observed in the RBD‐TC‐Sulfo‐Cy5 conjugate, showing that it may be a universal phenomenon. We therefore explored whether HSA‐TC‐proDOX possessed similar properties. The cancer cellular uptake of HSA‐TC‐proDOX was assessed in HeLa cells using flow cytometry. Another HSA and aldoxorubicin conjugate with doxorubicin modified to the free cysteine at position 34 (HSA‐Cys34‐proDOX) was prepared as a control. As shown in Figure 2d, the uptake of HSA‐TC‐proDOX was higher than that of HSA‐Cys34‐proDOX, consistent with the data obtained from HSA‐TC‐Sulfo‐Cy5 and RBD‐TC‐Sulfo‐Cy5. The tumor targeting ability of HSA‐TC‐proDOX was further assessed in an MDA‐MB‐231 xenograft model. As shown in Figure 2e, accumulation of HSA‐TC‐proDOX in tumors was also observed.

In exploring the mechanism of enhanced cellular uptake, we found that HSA‐TC‐proDOX formed nanoparticles with an average diameter of approximately 21 nm, as indicated by transmission electron microscopy (TEM) and dynamic light scattering (DLS) analysis (Figure 2f and Figure S7). Looking into the synthesis procedures (detailed in Section 5.2), we reasoned that the introduced sulfhydryl groups were much more likely to tend to form intermolecular disulfide bonds. Because, first, the aqueous buffer used was not deoxidized, which provided an oxidative environment, and the pH of the reaction buffer was slightly basic (pH 7.5), which both favored the formation of disulfide bonds via the mechanism of air oxidation.^32^ In addition, in the conjugation step, dimethyl sulfoxide (DMSO) was added as a cosolvent. It has been reported that DMSO can serve as an oxidation reagent to facilitate the formation of disulfide bonds.^33^ Moreover, sulfhydryl groups were installed on the surface exposing lysine residues, which made them accessible to each other and thus provided favorable conditions for disulfide bond formation. Therefore, we speculated that the excess amount of free sulfhydryl groups present on HSA may form intramolecular or intermolecular disulfide bonds during the overnight reaction. Based on this assumption, we further analyzed the particle sizes of HSA‐TC‐Sulfo‐Cy5 and RBD‐TC‐Sulfo‐Cy5 using TEM, and the images showed that HSA‐TC‐Sulfo‐Cy5 and RBD‐TC‐Sulfo‐Cy5 also formed nanometric particles with average sizes of 20 and 8 nm, respectively (Figures S8 and S9).

Hence, we proposed the mechanism of nanoparticle assembly during the thiolation‐and‐conjugation approach via the formation of intermolecular disulfide bonds (Figure 2g). To prove the presence of intermolecular disulfide bonds, reducing and nonreducing SDS–PAGE was performed. As shown in Figure S10, larger molecular weight bands clearly existed under nonreducing conditions and disappeared under reducing conditions, proving the presence of intermolecular disulfide bonds. Therefore, these data further supported the existence of nanometric particles. To better illustrate the nanoscale structure of the conjugate, HSA‐TC‐proDOX is referred to hereafter as NanoAlb‐proDOX.

Previous research has shown that the cellular uptake of gold nanoparticles increases with increasing particle size.^34^ Our own previous study also proved that nanosized protein nanoparticles facilitate cellular uptake.^35^ Therefore, the formation of albumin nanoparticles should be a reasonable explanation for the enhanced cellular uptake of NanoAlb‐proDOX.

Nanomaterials can be internalized by target cells through different mechanisms. For example, micrometer‐sized particles usually enter target cells through phagocytosis or micropinocytosis, while nanometer‐sized particles typically enter target cells through other types of endocytosis, such as clathrin‐dependent, caveolin‐dependent or receptor‐mediated endocytosis.^36^ The nanoparticles described in this study may undergo the mechanism for nanometer‐sized particles. Data acquired for HSA‐TC‐Sulfo‐Cy5 and RBD‐TC‐Sulfo‐Cy5, which were both prepared through the thiolation‐and‐conjugation method, showed that both displayed enhanced cellular uptake by cancer cells. However, the two proteins enter cells through different receptors. We therefore reasoned that the enhanced cellular uptake may be independent of their binding receptors. Clathrin‐dependent or caveolin‐dependent endocytosis may be the potential mechanism for the enhanced cellular uptake of albumin nanoparticles prepared by the thiolation‐and‐conjugation method. We therefore tested this hypothesis by using two inhibitors, Pistop 2 and genistein, which target clathrin‐ and caveolin‐dependent endocytosis,^37^ respectively. The results showed that both inhibitors reduced the endocytosis of HSA‐TC‐Cy5 in HeLa cells, indicating that the conjugate may be internalized through both endocytosis pathways (Figure S11).

### 2.3 Selectivity and toxicity of NanoAlb‐proDOX

**FIGURE 3:**
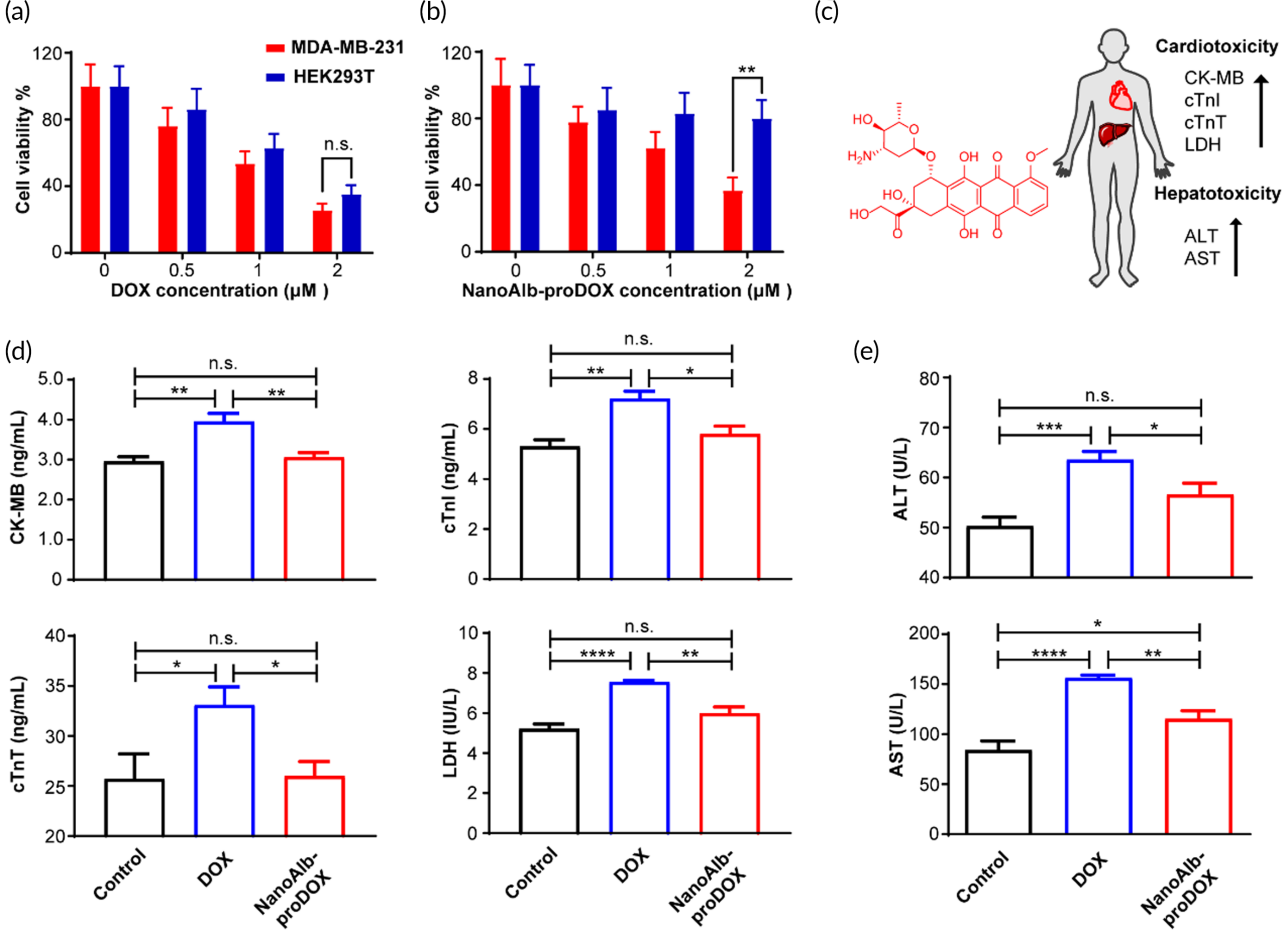
Analysis of in vitro cytotoxicity and in vivo cardiotoxicity and hepatotoxicity of NanoAlb‐proDOX. (a) Dose‐dependent cytotoxicity of doxorubicin toward MDA‐MB‐231 cancer cells and HEK293T noncancer cells. No significant cytotoxicity differences between the two cell lines were observed. *n* = 3 and represents three technical replicates. (b) Dose‐dependent cytotoxicity of NanoAlb‐proDOX to MDA‐MB‐231 cancer cells and HEK293T noncancer cells. Significantly higher cytotoxicity to MDA‐MB‐231 cancer cells was observed at higher concentrations. *n* = 3 and represents three technical replicates. Cells were treated with the indicated drugs for 24 h in (a) and (b) prior to the cell viability test. (c) Schematic representation of the cardiotoxicity and hepatotoxicity induced by doxorubicin and related upregulated protein markers. (d) Serum concentrations of cardiotoxicity markers. *n* = 5 mice. (e) Serum concentrations of hepatotoxicity markers. *n* = 5 mice. Serum markers were detected using ELISA. Doxorubicin induced higher concentrations of cardiotoxicity and hepatotoxicity markers, while NanoAlb‐proDOX showed no obvious cardiotoxicity or hepatotoxicity. Data are presented as the mean ± SEM. n.s. not significant, *p < 0.05, **p < 0.01, ***p < 0.001, ****p < 0.0001

The selective cytotoxicity of anticancer drugs to cancer cells is critical to define their safety profiles. We therefore first evaluated the cytotoxicity of NanoAlb‐proDOX in vitro. When internalized by cancer cells, doxorubicin localizes to the nucleus to cause DNA damage (Figure S12). Similarly, when incubated with cancer cells, NanoAlb‐proDOX also localized doxorubicin to the cell nucleus (Figure S12). We next further assessed the cytotoxicity of doxorubicin and NanoAlb‐proDOX to MDA‐MB‐231 cancer cells and noncancer HEK293T cells in vitro. Cells were treated with multiple concentrations of either drug. At each concentration, the cytotoxicity of doxorubicin to both cell lines was similar (Figure 3a). At higher concentrations, NanoAlb‐proDOX showed higher cytotoxicity to MDA‐MB‐231 cells (37% cell viability for MDA‐MB‐231 vs. 80% cell viability for HEK293T; Figure 3b). These results indicated that NanoAlb‐proDOX may possess more selective cytotoxicity to cancer cells than doxorubicin, probably due to the pH‐sensitive release of doxorubicin from NanoAlb‐proDOX, as it has been reported that cancer cells have a lower lysosomal pH than normal cells (pH 3.8–4.7 vs. pH 4.5–6).^38^

Chemotherapy drugs usually lead to adverse side effects, and doxorubicin induced obvious cardiotoxicity and hepatotoxicity during clinical application (Figure 3c); therefore, we evaluated the toxicity of NanoAlb‐proDOX in vivo. Either NanoAlb‐proDOX or doxorubicin was administered to mice at an equivalent dosage of 15 mg/kg doxorubicin intraperitoneally. Forty‐eight hours later, the mice were sacrificed, and the serum concentrations of the cardiotoxicity markers creatine kinase‐MB (CK‐MB), cardiac troponin I (cTnI), cardiac troponin T (cTnT), and lactate dehydrogenase (LDH) (Figure 3d) and the serum concentrations of the hepatotoxicity markers alanine transaminase (ALT) and aspartate aminotransferase (AST) (Figure 3e) were measured using enzyme‐linked immunosorbent assays (ELISA). Doxorubicin induced absolutely higher levels of these markers than in the control group, consistent with the clinically reported adverse side effects of doxorubicin, namely, cardiotoxicity and hepatotoxicity. However, only slightly higher (for AST) or even no obvious change (for the other five markers) was observed for the NanoAlb‐proDOX‐treated group, revealing reduced side effects and better safety profiles for NanoAlb‐proDOX in vivo.

### 2.4 Antitumor activity of NanoAlb‐proDOX

**FIGURE 4:**
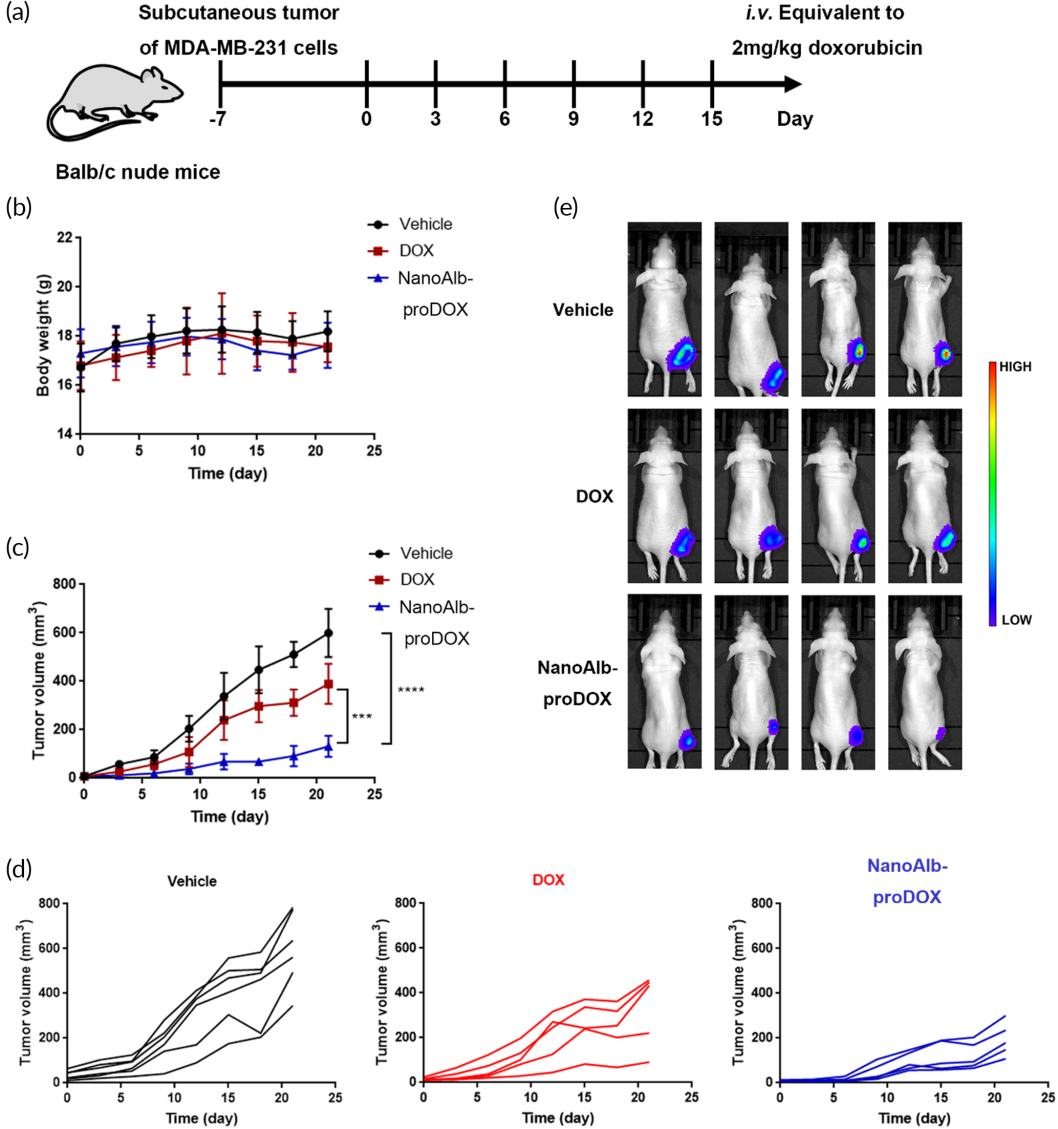
Antitumor activity of NanoAlb‐proDOX in breast cancer xenografts. (a) Schematic representation of the design of animal experiments. Drugs were administered intravenously every 3 days for a total of six doses. (b) Body weight curves of the treated mice. Body weights were measured every 3 days. (c) Average tumor volume curves of the treated mice. Tumor volumes were measured every 3 days. Significantly reduced tumor proliferation was observed in mice treated with NanoAlb‐proDOX. (d) Individual tumor growth curves of the mice treated with Vehicle, DOX or NanoAlb‐proDOX. (e) Representative bioluminescence images of the mice posttreatment. Images were taken at Day 21. The weakest bioluminescence signal was observed in mice treated with NanoAlb‐proDOX. Data are presented as the mean ± SEM. *n* = 6 mice for the vehicle group and *n* = 5 mice for the DOX and NanoAlb‐proDOX groups. ***p < 0.001, ****p < 0.0001

We next tried to evaluate the antitumor activity of NanoAlb‐proDOX in vivo. Due to the in vivo targeting of MDA‐MB‐231 triple‐negative breast cancer xenografts and enhanced uptake of NanoAlb‐proDOX by cancer cells, we first established MDA‐MB‐231 xenografts in female Balb/c nude mice. Mice were inoculated with MDA‐MB‐231 cancer cells in the right flank approximately 7 days prior to treatment and randomly divided into three groups (Vehicle, DOX, and NanoAlb‐proDOX groups) with tumor volumes of approximately 50–100 mm^3^. Each group was intravenously administered saline (Vehicle), 2 mg/kg doxorubicin (DOX) or NanoAlb‐proDOX (NanoAlb‐proDOX, equivalent to 2 mg/kg doxorubicin) every 3 days for a total of six doses (Figure 4a). Tumor volumes and body weights were measured every 3 days after the start of the treatment. As shown, the body weights of all three groups were not greatly affected at this dosage (Figure 4b). In contrast, tumor growth was strongly inhibited by treatment with NanoAlb‐proDOX (78% tumor suppression), and only moderate inhibition was observed for the group treated with doxorubicin (35% tumor suppression, Figure 4c). Individual tumor volumes were plotted and also showed that mice treated with NanoAlb‐proDOX exhibited more shrunken tumors (Figure 4d). After treatment, bioluminescence imaging (MDA‐MB‐231 cells stably transfected with the firefly luciferase gene) was performed to further confirm the antitumor activity of NanoAlb‐proDOX. As shown in Figure 4e, mice treated with NanoAlb‐proDOX had the weakest bioluminescence intensity, indicating the strongest antitumor activity of NanoAlb‐proDOX. All these data proved that NanoAlb‐proDOX possessed better antitumor activity than doxorubicin against triple‐negative breast cancer xenografts.

Breast cancer is the most common type of tumor in women.^39^ While effective treatment and good prognosis can be expected for breast cancer patients, the treatment and prognosis are usually not satisfying for triple‐negative breast cancer subtype patients, who make up 15%–20% of all breast cancer patients.^40^ The NanoAlb‐proDOX introduced here showed great anti‐triple negative breast cancer activity in a mouse xenograft model, which may make NanoAlb‐proDOX an effective alternative for the treatment of triple‐negative breast cancer.

In addition to breast cancer, ovarian cancer is the sixth most common and most lethal cancer in women.^41^ Ovarian cancer develops ascites during the late stage,^42^ which exacerbates the patient's condition. Therefore, we next validated whether NanoAlb‐proDOX can address the situation of ascites in ovarian cancer. We constructed an ascites model of ovarian cancer using the ID8‐Luc cell line (a mouse ovarian cancer cell line stably transfected with firefly luciferase, Figure S13A). ID8‐Luc cells were directly injected into the peritoneal cavity of female C57BL/6 mice to allow tumor dissemination and ascites formation in approximately 4 weeks. The establishment of tumors in the peritoneal cavity was monitored using in vivo live animal bioluminescence. Available xenograft mice were divided into three groups, which were administered PBS, 1.5 mg/kg doxorubicin (DOX) or NanoAlb‐proDOX (NanoAlb‐proDOX, equivalent to 1.5 mg/kg doxorubicin) intravenously every 3 days for a total of five doses (Figure S13A). The body weights of the mice in each group were monitored every 3 days during the administration period. As indicated, the body weights of the mice in the PBS and DOX groups were slightly higher, mainly due to heavily developed ascites masses, while the body weights of the mice in the NanoAlb‐proDOX‐treated group remained stable with reduced ascites masses (Figure S13B). The progression of ascites and tumor dissemination was monitored by in vivo bioluminescence imaging (Figure S13C). Images showed that both groups of mice treated with either doxorubicin or NanoAlb‐proDOX displayed reduced bioluminescence signals compared with PBS‐treated mice, indicating the effectiveness of both drugs in the ascites xenograft model. Quantitative analysis of the bioluminescence imaging data from Day 0 and Day 14 revealed that doxorubicin treatment still resulted in slight tumor proliferation, while NanoAlb‐proDOX treatment led to stable or even slightly reduced tumor proliferation (Figure S13D). Afterward, survival of the mice was monitored. Survival curves revealed prolonged median survival times for both doxorubicin‐ and NanoAlb‐proDOX‐treated mice, with NanoAlb‐proDOX treatment leading to greater benefits (median survival times: 100 days for NanoAlb‐proDOX versus 81 days for doxorubicin, Figure S13E).

In brief, the NanoAlb‐proDOX invented here showed improved antitumor activity in both triple‐negative breast cancer and ovarian ascites xenograft models. Taking into consideration its selective cytotoxicity toward cancer cells in vitro and reduced side effects in vivo, NanoAlb‐proDOX may be a good choice for cancer therapy.

### 2.5 Synergy with immune checkpoint blockade

**FIGURE 5:**
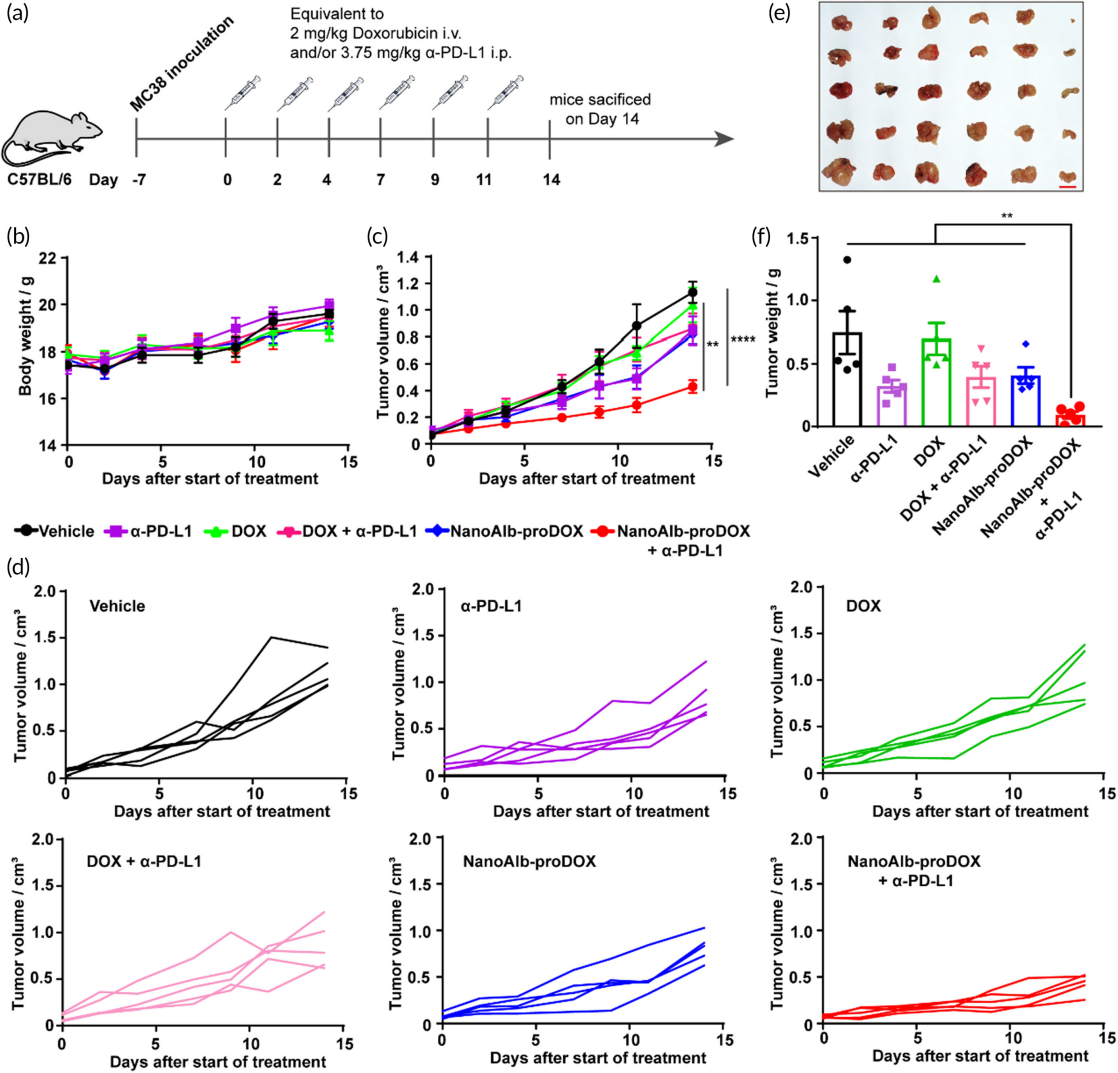
NanoAlb‐proDOX synergized with immune checkpoint blockade. (a) Schematic representation of the design of animal experiments. Drugs were administered on the indicated days intravenously or intraperitoneally and sacrificed on Day 14. (b) Body weight curves. (c) Average tumor volume curves. Mice were randomly divided into six groups. Body weights and tumor volumes were measured every 2 or 3 days from Day 0 to Day 14. (d) Individual tumor growth curves of the six groups of mice. (e) Images of the tumor tissues. Scale bar: 1 cm. (f) Weights of the collected tumor tissues; (e) and (f) used the same group labels. Data are presented as the mean ± SEM. *n* = 5 mice for each group. **p < 0.01, ****p < 0.0001

Although immune checkpoint blockade has been proven powerful to battle cancer, the overall responses are low,^43^, ^44^, ^45^ and great improvement is therefore still desired. One promising solution is combining chemotherapy with immune checkpoint inhibitors.^13^ The NanoAlb‐proDOX invented in the manuscript showed improved safety profiles and superior antitumor activity; therefore, we combined NanoAlb‐proDOX treatment with anti‐PD‐L1 antibody (α‐PD‐L1) inhibition and validated its efficacy in vivo. Colorectal cancer xenograft models were constructed with MC38 cells inoculated into the right flanks of mice. Prior to therapeutic evaluation, the time‐course accumulation of both NanoAlb‐proDOX and α‐PD‐L1 in tumors was assessed in MC38 xenografts. As shown in Figure S14, both therapeutic proteins displayed good accumulation in tumors with retention times over 72 h. A therapeutic evaluation protocol was therefore designed (Figure 5a). Mice were randomly divided into six groups (Vehicle, α‐PD‐L1, DOX, NanoAlb‐proDOX, DOX + α‐PD‐L1, and NanoAlb‐proDOX + α‐PD‐L1 groups) when tumor volumes reached 50–100 mm^3^. DOX and NanoAlb‐proDOX were administered intravenously, and α‐PD‐L1 was administered intraperitoneally at dosages of 2 mg/kg doxorubicin or 3.75 mg/kg antibody every 2 days for a total of six doses. For evaluation, the body weights of the mice and tumor volumes were measured at the indicated times. As shown, the body weights remained stable in all six groups (Figure 5b). Average tumor volumes were plotted (Figure 5c). As indicated, doxorubicin treatment only led to minimal inhibition of tumor proliferation, while NanoAlb‐proDOX treatment resulted in better inhibition (4.9% vs. 14.9% suppression), which was consistent with the results obtained in triple‐negative breast cancer and ovarian cancer ascites models. Treatment with the α‐PD‐L1 antibody showed some benefits in tumor inhibition (9.8% suppression). In combination, doxorubicin and α‐PD‐L1 showed no obvious synergistic effects and did not promote tumor growth inhibition, reflecting the limited efficacy of doxorubicin. Nevertheless, the combination of NanoAlb‐proDOX and α‐PD‐L1 showed good synergy and exhibited dramatic tumor growth inhibition (55.3% suppression), coinciding with the superior antitumor activity of NanoAlb‐proDOX. Individual tumor volume curves were also plotted for all six groups (Figure 5d). Synergistic tumor inhibition was observed in all five mice in the NanoAlb‐proDOX and α‐PD‐L1 combination group.

Afterward, the mice were sacrificed, and tumor tissues were collected, weighed, and imaged. Consistent with the tumor volumes measured, the combination of NanoAlb‐proDOX and α‐PD‐L1 led to the smallest tumor tissues (Figure 5e,f), further confirming the synergistic effects of NanoAlb‐proDOX and α‐PD‐L1. To examine safety considerations, the major organs of the mice were also collected and subjected to hematoxylin eosin (H&E) staining. Morphology of the nucleolus, nucleocytoplasmic ratio and tissue structure showed no obvious changes in all groups, indicating no obvious organ damage under these experimental settings (Figure S15).

### 2.6 Enhanced tumor infiltration of CD8 ^+^ T cells

**FIGURE 6:**
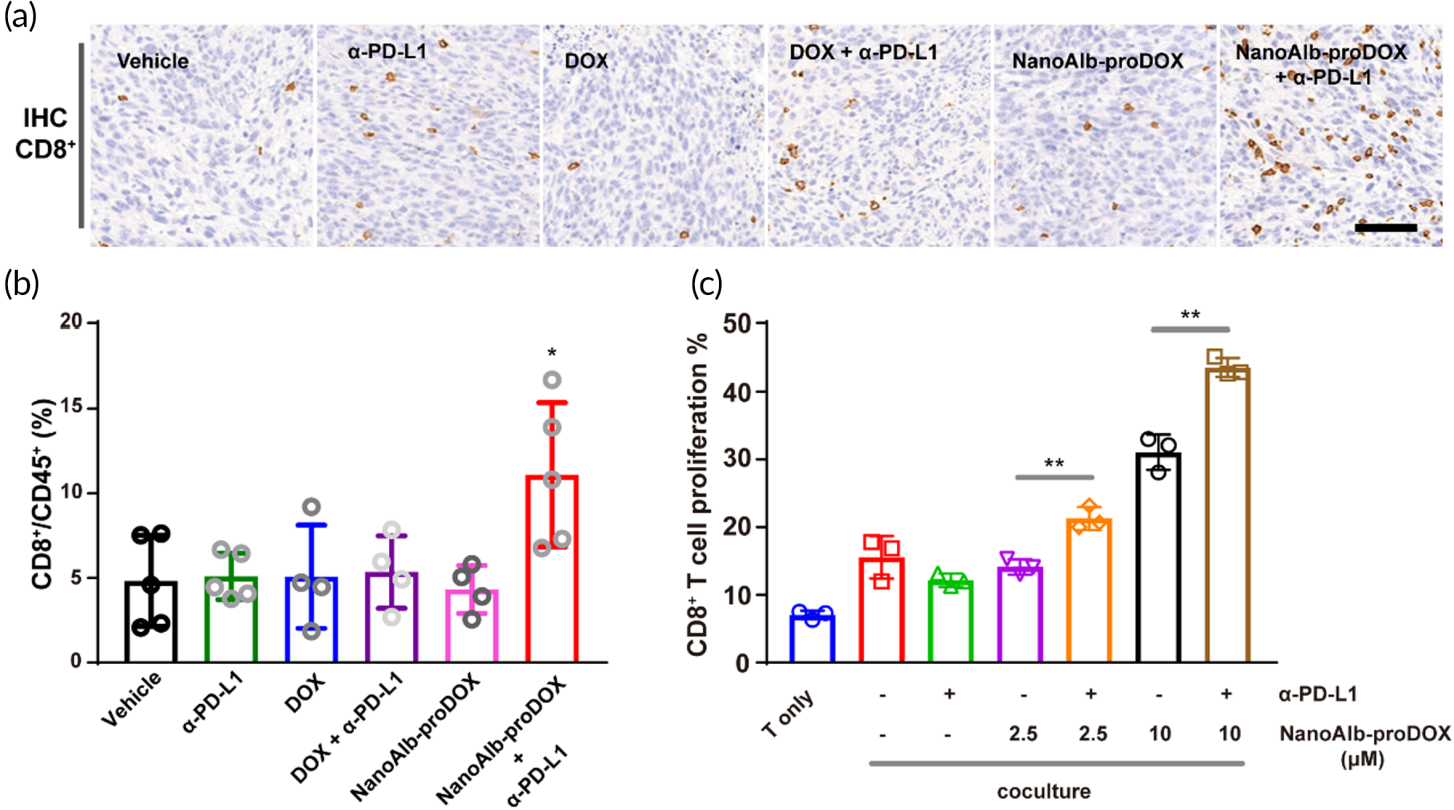
(a) Immunohistochemical (IHC) staining of infiltrated CD8^+^ T cells in tumor tissues. Scale bar: 100 μm. (b) Quantitative analysis of CD8^+^ T cells in tumor tissues from flow cytometry data. Tumor tissues were digested and resuspended. Single and viable cells were gated using flow cytometry. The CD8^+^ cell population in the CD45^+^ cell population was assessed. *n* = 4 or 5 mice. Asterisks indicate significance for the NanoAlb‐proDOX plus α‐PD‐L1 group against all other groups. (c) In vitro coculture assay to validate the proliferation of CD8^+^ T cells. CD8^+^ T cells were labeled with CFSE to monitor the proliferation of T cells. *n* = 3 and represents three technical replicate cell culture wells. Data are presented as the mean ± SEM. *p < 0.05, **p < 0.01

The efficacy of immune checkpoint blockade is limited by the infiltration and activation of T cells in tumors, especially cytotoxic CD8^+^ T cells. NanoAlb‐proDOX had a synergistic effect with α‐PD‐L1 treatment, so we asked whether this synergy was related to different T‐cell infiltrations. Therefore, the tumor tissues collected above were subjected to immunohistochemistry (IHC) analysis. Tumor tissue slices were stained with anti‐mouse CD8 antibody (Figure 6a). As shown in Figure 6a, the infiltration of CD8^+^ cells into the untreated tumor tissue was rare, as shown by the IHC images from the Vehicle group. Monotherapies with α‐PD‐L1, doxorubicin or NanoAlb‐proDOX and combination therapy with α‐PD‐L1 and doxorubicin barely increased CD8^+^ T‐cell infiltration. However, combination therapy with α‐PD‐L1 and NanoAlb‐proDOX considerably increased the infiltration of CD8^+^ T cells. The IHC data were quantitatively analyzed (Figure S16), which also revealed that the combination of α‐PD‐L1 and NanoAlb‐proDOX significantly increased the infiltration of CD8^+^ T cells.

To confirm the T‐cell infiltration results obtained from IHC, tumor tissues were also digested and resuspended in single cells for flow cytometry analysis. Cells were stained with viability dye, anti‐mouse CD45 and anti‐mouse CD8 antibodies. Single and live cells were gated to quantify the proportions of CD8^+^ cells among CD45^+^ cells (Figure S17). The flow cytometry data are quantitatively summarized in Figure 6b, which shows that the combination of NanoAlb‐proDOX and α‐PD‐L1 led to an approximate 2‐fold increase in CD8^+^ T cells in tumor‐infiltrated leukocytes compared to the other groups. IHC and flow cytometry data both proved that CD8^+^ T‐cell infiltration in tumors was promoted by combination therapy with NanoAlb‐proDOX and α‐PD‐L1, supporting the utilization of NanoAlb‐proDOX as a possible solution to strengthen the efficacy of immune checkpoint blockade.

To further support the findings from IHC and flow cytometry, we also conducted an in vitro coculture assay using purified CD8^+^ T cells to validate whether cotreatment with NanoAlb‐proDOX and α‐PD‐L1 stimulates the proliferation of CD8^+^ T cells.^46^ CD8^+^ T cells were cocultured with MC38 cells with the addition of α‐PD‐L1, NanoAlb‐proDOX, or both. The proliferation of CD8^+^ T cells was monitored by measuring the prelabeled CFSE fluorescence in CD8^+^ T cells. As summarized in Figure 6c, CD8^+^ T‐cell proliferation was enhanced by NanoAlb‐proDOX in a concentration‐dependent manner. The combination of NanoAlb‐proDOX and α‐PD‐L1 further increased the proliferation of CD8^+^ T cells. This dataset provided the insight that the enhanced CD8^+^ T‐cell infiltration in vivo may result from the enhanced CD8^+^ T‐cell proliferation costimulated by NanoAlb‐proDOX and α‐PD‐L1.

The activation state of the infiltrated T cells is another important factor influencing the efficacy of immune checkpoint blockade. We therefore evaluated T‐cell activation by measuring the levels of interferon‐γ (IFN‐γ) and interleukin 2 (IL‐2) in tumor tissues using an enzyme‐linked immunosorbent assay (ELISA). Consistent with the T‐cell infiltration results, the levels of both cytokines were increased in the NanoAlb‐proDOX and α‐PD‐L1 combination group (Figure S18), providing evidence for the activation of infiltrated T cells.

In brief, the results proved that combination therapy with NanoAlb‐proDOX and α‐PD‐L1 enhanced the tumor infiltration of T cells and facilitated their activation. Using an in vitro coculture assay, we provided evidence that the enhanced infiltration may result from elevated T‐cell proliferation stimulated by the coadministration of NanoAlb‐proDOX and α‐PD‐L1. These data supported the utilization of NanoAlb‐proDOX as a costimulator for immune checkpoint blockade.

## 3 DISCUSSION

In this study, we described the validation of a thiolation‐and‐conjugation method to prepare protein and drug conjugates. The versatility of this method was first demonstrated on HSA and the recombinant receptor‐binding domain of SARS‐CoV‐2 by preparing their conjugates with fluorophores (Figure 1 and Figure S4). The thiolation‐and‐conjugation method did not affect the in vivo tumor targeting ability of albumin in a human triple‐negative breast cancer model (Figure 1c). Moreover, the conjugates prepared through this method possessed enhanced cancer cellular uptake in vitro (Figure 1d and Figure S4), suggesting that the protein–drug conjugates prepared via this strategy may be useful in cancer therapy.

As a proof of concept, we prepared an albumin doxorubicin conjugate with the thiolation‐and‐conjugation method (Figure 2). In addition to the conjugation method, the doxorubicin drug molecule was designed to contain an acid‐sensitive linker to facilitate the specific release of the drug molecule from the conjugate (Figure 2a,c). As part of the validation, we revealed that the albumin and doxorubicin conjugate (HSA‐TC‐proDOX) exhibited good tumor targeting in vivo and enhanced cancer cellular uptake in vitro (Figure 2d,e). In exploring the mechanism behind the enhanced cancer cellular uptake of the prepared conjugates, we thought that the native receptors of the proteins may play very limited roles, as albumin and RBD bind to different receptors (e.g., SPARC/gp60 for albumin and ACE2 for RBD). Using TEM and DLS technologies, we discovered that the HSA‐TC‐proDOX, HSA‐TC‐Sulfo‐Cy5 and RBD‐TC‐Sulfo‐Cy5 all formed a nanosized structure (Figure 2f, Figure S7, S8 and S9), and we proposed a possible mechanism involving the formation of intermolecular disulfide bonds (Figure 2g). We therefore speculated that the nanoparticle structure may be a key reason for the enhanced cellular uptake, as nanoparticles tend to accumulate in cells. To further determine the endocytosis pathway of the conjugate prepared via the thiolation‐and‐conjugation method, we performed an endocytosis inhibition assay with clathrin and caveolin inhibitors. The results showed that the cellular uptake of the albumin conjugate was reduced by both inhibitors, indicating that the conjugate may adopt both clathrin‐dependent and caveolin‐dependent endocytosis pathways (Figure S11).

To validate the properties of the NanoAlb‐proDOX conjugate, we showed that NanoAlb‐proDOX preferentially killed cancer cells in vitro (Figure 3a) and had reduced side effects in vivo compared with doxorubicin (Figure 3b–d). These data indicated that NanoAlb‐proDOX could become a safer antitumor drug. The effectiveness of NanoAlb‐proDOX was validated in two different xenograft models, in which both revealed that NanoAlb‐proDOX was more effective than doxorubicin (Figure 4 and Figure S13). In another combination therapy trial, NanoAlb‐proDOX was coadministered with α‐PD‐L1 (Figure 5). The results showed that the two molecules induced significant synergistic effects and greatly inhibited tumor proliferation in a colorectal cancer xenograft model (Figure 5). In exploring the mechanism behind the synergistic effects, we found that the tumor infiltration of CD8^+^ T cells was enhanced (Figure 6a,b). With an in vitro assay, we provided evidence that the enhanced infiltration may be a result of enhanced CD8^+^ T‐cell proliferation, which was stimulated by the coadministration of NanoAlb‐proDOX and α‐PD‐L1 (Figure 6c). These data indicated that NanoAlb‐proDOX can be a costimulator of immune checkpoint blockade. In addition, with a preliminary trial, we found that NanoAlb‐proDOX induced a small amount of tumor cell pyroptosis in vitro (data not shown in this manuscript, but available if required). It has also been reported in the literature that cytotoxic lymphocytes induce tumor cell pyroptosis, and tumor cell pyroptosis in turn induces T‐cell infiltration.^47^, ^48^ We therefore reasoned that in combination, NanoAlb‐proDOX and α‐PD‐L1 may induce potent tumor cell pyroptosis, which then induces cytotoxic CD8^+^ T‐cell infiltration/proliferation. This hypothesis will be tested in our following study.

## 4 CONCLUSIONS

In summary, we reported the validation of a generally applicable thiolation‐and‐conjugation approach to generate protein–drug conjugates. The resulting protein–drug conjugates were not affected by the conjugation method in terms of the tumor targeting ability in vivo and exhibited enhanced cancer cellular uptake. As a proof of concept of the utility of the method, an albumin–doxorubicin prodrug conjugate NanoAlb‐proDOX was prepared. NanoAlb‐proDOX self‐assembled into nanoparticles, probably via the formation of intermolecular disulfide bonds, which were believed to be responsible for its enhanced cancer cellular uptake. NanoAlb‐proDOX exhibited selective cytotoxicity toward cancer cells in vitro and reduced side effects in vivo. In two mouse tumor xenograft models, it showed superior antitumor activity to the parent chemotherapy drug doxorubicin. In a combination therapy trial, NanoAlb‐proDOX synergistically elevated the therapeutic efficacy of immune checkpoint blockade with enhanced T‐cell tumor infiltration and activation. All the data indicated that the thiolation‐and‐conjugation method can serve as a general strategy to prepare protein and drug conjugates, and the proof‐of‐concept albumin–doxorubicin conjugate can be a good choice of new antitumor drug, which showed superior activity and may serve as a costimulator of immune checkpoint blockade.

## 5 MATERIALS AND METHODS

### 5.1 Materials

NHS‐Cy5.5 (Cat. # 47020), NHS‐Sulfo‐Cy5 (Cat. # 43320), MAL‐Sulfo‐Cy5 (Cat. # 43380), NHS‐Cy5 (Cat. # 43020), and MAL‐Cy5 (Cat. # 43080) were purchased from Lumiprobe. d‐Luciferin potassium salt (Cat. # 50227) and doxorubicin (Cat. # D1515) were purchased from Sigma‐Aldrich. SPDP (Cat. # HY‐100216), aldoxorubicin (Cat. # HY‐16261), Pistop 2 (Cat. # HY‐115604), Genistein (Cat. # HY‐14596), and GSH (Cat. # HY‐D0187) were purchased from MedChemExpress. The PD‐10 desalting column was purchased from GE (Cat. # 17085101). HSA and anti‐PD‐L1 antibody were kindly gifts from Zhejiang Hisun Pharmaceutical Co., Ltd. Anti‐CD8 antibody was purchased from Cell Signaling Technology (Cat. # 98941S). RBD protein was expressed and purified from Pichia pastoris by our collaborators.^35^ All mice (BALB/c nude and C57BL/6) were purchased from Charles River.

### 5.2 Methods

#### 5.2.1 Synthesis of albumin conjugates and NanoAlb‐proDOX conjugate

HSA was dissolved in PBS‐EDTA buffer (20 mM sodium phosphate, 150 mM NaCl, 1 mM EDTA, pH 7.5) to a concentration of 200 μM. SPDP stock solution was prepared in DMSO. To initiate the reaction, 50 μM HSA and 1 mM SPDP were gently shaken for 2 h at room temperature in PBS‐EDTA buffer. After the reaction, the excess SPDP was removed by flowing through a PD‐10 column (GE). Then, 23 mg/ml DTT in acetate buffer (100 mM sodium acetate buffer, 100 mM NaCl, pH 4.5) was added to SPDP‐labeled HSA in PBS‐EDTA buffer with a final concentration of HSA of approximately 100 μM (the ratio of acetate buffer and PBS‐EDTA buffer was kept at 1:2 to keep the pH of the reaction system relatively acidic, preventing the native and intracellular disulfide bonds from being affected by DTT reduction). The reaction proceeded at room temperature for 1 h, and the excess DTT was removed by flowing through a PD‐10 column (GE). The reduced HSA solution was then allowed to react with 100 μM aldoxorubicin (AlDOX, prediluted with 20% of total reaction volume of DMSO) with a final HSA concentration of 20 μM in PBS‐EDTA buffer. The mixture was gently shaken overnight at room temperature. Finally, excess AlDOX was removed by flowing through a PD‐10 column (GE). NanoAlb‐proDOX solution was stored in PBS buffer and preserved at −80°C. Cyanine 5 albumin/RBD conjugates (HSA‐TC‐Sulfo‐Cy5/HSA‐TC‐Sulfo‐Cy5/RBD‐TC‐Sulfo‐Cy5) were synthesized following the same approach. Conjugation of albumin/RBD with NHS‐Sulfo‐Cy5/NHS‐Cy5 or conjugation at cysteine 34 with aldoxorubicin was performed by directly mixing the two components followed by PD‐10 column purification.

#### 5.2.2 Characterization of NanoAlb‐proDOX

To determine the conjugation efficiency of HSA‐proDOX, a method that plots the fluorescence intensity of doxorubicin was adopted. A standard curve was established by measuring the fluorescence of doxorubicin with excitation at 485 nm and emission at 590 nm. The amount of doxorubicin conjugated to albumin was determined by fitting its fluorescence to the standard curve. The concentration of HSA‐proDOX was determined by a BCA Protein Assay Kit. The efficacy of the conjugation was finally calculated and defined as the concentration of doxorubicin/concentration of protein, and the number was determined to be 3.0. To determine the conjugation efficiency of fluorophores, fluorophore concentrations were measured using Nanodrop 2000 with the Protein & Label program. All samples were dissolved in PBS buffer.

For DLS, the proteins were dissolved at a concentration of ~0.1 mg/ml in phosphate buffered saline (PBS) buffer. The measurement was performed on a Zetasizer Nano ZS‐90 (Malvern). For TEM, NanoAlb‐proDOX specimens were stained with phosphotungstic acid. Briefly, 10 μl of protein sample (0.2 mg/ml) was applied to the TEM grid and allowed to settle for 10 min. Excess fluid was wicked off with clean filter paper. Samples were stained with 2% (w/v) phosphotungstic acid for 3 min. Excess fluid was wicked off with clean filter paper, and the grid was further air dried for 10 min. TEM images were captured with JEM‐2100F microscope (JEOL) or H‐7650 microscope (Hitachi).

To characterize the pH‐dependent release of doxorubicin from NanoAlb‐proDOX, an ultrafiltration‐based method was adopted. First, NanoAlb‐proDOX (final concentration 1 mg/ml) was incubated at room temperature with buffer pH adjusted to 7.5 and 5.5. At the indicated times, the incubation solution was moved to an ultrafiltration unit with a 10 kDa cutoff. After centrifugation, the solution that passed through the ultrafiltration filter was collected. The doxorubicin concentration in the pass‐through solution was determined and defined as the amount of doxorubicin released from NanoAlb‐proDOX.

### 5.3 Cell culture

MDA‐MB‐231 cells were obtained from PerkinElmer. HeLa, HEK293T, MC38, and ID8 cells were obtained from ATCC. MDA‐MB‐231‐Luc and ID8‐Luc cells were produced using a lentivirus‐based vector harboring firefly luciferase. Briefly, HEK293T cells were first transfected with psPAX2, pMD2. G and pLV‐luci (Inovogen Tech. Co. Catalog No. VL3612) plasmids to pack the target lentivirus. Lentiviruses harboring the firefly luciferase genes were acquired from cell culture medium supernatant collected 48, 72, 96 h after plasmid transfection and concentrated using an ultrafiltration centrifugal filter unit (Millipore, Catalog No. UFC903096, 30 kDa cutoff). Then, MDA‐MB‐231 and ID8 cells were transfected with packed lentivirus supplemented with 10 μg/mL polybrene (Shanghai Yuanye Bio‐Technology Co., Ltd. Catalog No. S24797). Cell culture medium was replaced by fresh medium 24 h later, and luciferase‐positive cells were selected with 20 μg/ml puromycin (Solarbio, Catalog No. P8230) over a 2‐week period. The expression of luciferase was validated using the PerkinElmer IVIS III imaging system. All cell culture‐related reagents were purchased from Gibco. MDA‐MB‐231, HeLa, HEK293T, and ID8 cells were cultured in DMEM supplemented with 10% fetal bovine serum (FBS) and 1% penicillin/streptomycin at 37°C under 5% CO_2_. MC38 cells were cultured in RPMI 1640 culture medium supplemented with 10% fetal bovine serum (FBS) and 1% penicillin/streptomycin at 37°C under 5% CO_2_.

### 5.4 In vitro cytotoxicity assay

Cells were seeded in 96‐well plates at a density of 5000 cells per well. Twenty‐four hours later, NanoAlb‐proDOX or doxorubicin at the indicated concentrations predissolved in fresh medium was added to the cell culture. After 24 h of incubation, the cells were subjected to the MTT assay to determine cell viability and to calculate in vitro cytotoxicity.

### 5.5 Confocal microscopy imaging and flow cytometry analysis of cellular uptake

HeLa and MDA‐MB‐231 cells were cultured to approximately 90% confluence in 10 cm dishes. For confocal microscopy imaging, cells were seeded into eight‐well chamber slides with an inoculation ratio of 1:3. After incubating at 37°C for 24 h, the cells were treated with protein conjugates of the indicated concentrations in the manuscript. Three hours or 18 hlater, the cells were stained with Hoechst 33342. Florescence images were taken by Zeiss confocal microscopy (Zeiss LSM700) with Hoechst 33342 and doxorubicin channels. For flow cytometry analysis, HeLa and MDA‐MB‐231 cells were seeded in 24‐well plates with the same inoculation ratio. Twenty‐four hours later, the cells were treated with protein conjugates of the indicated concentrations in the manuscript. Three hours or 18 hlater, the HeLa or MDA‐MB‐231 cells were detached using 0.25% trypsin–EDTA and resuspended in PBS supplemented with 0.5% fetal bovine serum. The resuspended cells were then subjected to flow cytometry (Bio‐Rad S3e) to analyze the cellular uptake of the protein conjugates.

### 5.6 Inhibitor assay

HeLa cells were cultured to approximately 90% confluence in 10 cm dishes and then seeded into 24‐well plates with an inoculation ratio of 1:3 for overnight culture. When the cells reached 70%–80% confluence, the cell culture medium was replaced with serum‐free medium containing either 5 μM Pistop 2 or 100 μM Genistein. Cells were cultured for 30 min, and then 200 nM HSA‐TC‐Cy5 was added to the cell culture for another 1 h incubation. Then, the cells were detached from the culture plate using trypsin. Cells were resuspended in PBS (supplemented with 1% FBS), and cellular uptake of the conjugate was evaluated with flow cytometry.

### 5.7 Animal and tumor models

BALB/c nude mice and C57BL/6 mice aged 6–8 weeks, with an average weight of 20 g, were purchased from Charles River. Female mice were used in all experiments. Mice were raised in a specific pathogen‐free (SPF) animal house. For the construction of subcutaneous tumor models, mice were injected with 5x10^5^ MDA‐MB‐231 or MC38 cells in the right flank. For the MDA‐MB‐231 model, BALB/c nude mice were utilized. For the MC38 model, C57BL/6 mice were used. For the ascites model of ovarian cancer, 2 × 10^6^ ID8‐luciferase cells in 200 μl PBS were injected into the peritoneal cavity of female 6‐ to 8‐week‐old C57BL/6 mice. Tumor formation was monitored 3–4 weeks post‐injection by an in vivo imaging system (PerkinElmer IVIS III). Mice were randomly divided into the indicated groups and administered the indicated drugs (i.v. for DOX and NanoAlb‐proDOX and i.p. for anti‐PD‐L1 antibody). Tumor burdens were either evaluated with tumor volume (length * width * width)/2 for subcutaneous tumor models or measured by luminescence imaging for ascites models. All animal experiments were performed under the guidelines of the IACUC of Peking University Health Science Center (No. LA2020497).

For the animal experiment shown in Figure 4, drugs were administered every 3 days, on Days 0, 3, 6, 9, 12, and 15, for a total of six doses to five mice from each group. Tumor volumes and body weights were measured on Days 0, 3, 6, 9, 12, 15, 18, and 21. Bioluminescence images were taken at Day 21.

For the animal experiments related to Figures 5 and 6, drugs were administered every 2 or 3 days on Days 0, 2, 4, 7, 9, and 11 for a total of six doses with five mice from each group. Tumor volumes and body weights were measured on Days 0, 2, 4, 7, 9, 11, and 14. Mice were sacrificed on Day 14. Tumor tissue weights were measured, and images were taken. For IHC, tumor tissues from three individual mice were randomly selected. IHC was performed, and the data images were analyzed as described below. Procedures for flow cytometry analysis of the tumor‐infiltrated CD8‐positive cells are described below.

For the animal experiment related to Figure S10, drugs were administered every 3 days on Days 1, 4, 7, 10, and 13 for a total of five doses, with eight mice from each group. Body weights were measured on Days 1, 4, 7, 10, and 13. Bioluminescence images were taken at Day 0 and Day 14. Survival of the mice was monitored every day until Day 100.

In all animal experiments, doxorubicin and NanoAlb‐proDOX dosages were defined as the dosages of the administered doxorubicin molecules.

### 5.8 Detection of serum or tissue markers

Forty‐eight hours after doxorubicin or NanoAlb‐proDOX injection, the mice were sacrificed. Blood was collected from eyeballs, and serum was separated. Serum levels of ALT, AST, LDH, CK‐MB, cTnI, and cTnT were determined using ELISA. For the determination of IFN‐γ and IL‐2 in tumor tissues, the supernatant of the tumor tissue homogenates in PBS buffer was analyzed using ELISA.

### 5.9 Flow cytometry analysis of the infiltrated T cells

The mice shown in Figure 5 were sacrificed on Day 14 after a total of six doses, and the tumor tissues were recovered. Tissues were first cut into small pieces with scissors and washed with PBS. Then, collagenase/hyaluronidase was added to digest the tissues with supplement of DNase I. The digestion proceeded at 37°C with continuous shaking for 30 min before quenching with DMEM supplemented with FBS. The digested suspension then flowed through a cell strainer (100 μm). The recovered cells were washed and resuspended in PBS. Cells were first stained with a Zombie NIR™ Fixable Viability Kit (catalog no. 423106) and blocked with TruStain fcX™ anti‐mouse CD16/32 (catalog no. 101320). Subsequently, the cells were stained with FITC‐conjugated anti‐mouse CD45 antibody (Biolegend, catalog no. 103108) and PerCP/Cyanine5.5‐conjugated anti‐mouse CD8a antibody (Biolegend, catalog no. 100734). The stained cells were washed with PBS and ready for flow cytometry analysis after resuspension in PBS. Single and live cells with surface CD45 and CD8 markers were gated and quantitatively analyzed.

### 5.10 In vivo fluorescence and bioluminescence imaging

All in vivo images were taken with a PerkinElmer IVIS III system or IVScope 8200 (CLINX). For fluorescence imaging, proteins were first labeled with the indicated fluorophore (Cy5.5 or Cy5) and injected intravenously into mice. Images were taken in the cy5 channel. For bioluminescence imaging, mice were first injected intraperitoneally with d‐luciferin (1 mg/ml, 100 μl). Ten minutes later, images were taken with the bioluminescence settings.

### 5.11 In vitro CD8 ^+^ T‐cell proliferation

MC38 cells were seeded in 96‐well plates and cultured overnight to a confluence of approximately 80%. Primary CD8^+^ T cells were purified from C57BL mice and labeled with CFSE to measure proliferation. Prior to coculture, CD8^+^ T cells were stimulated with T‐cell TransAct™ (Miltenyi Biotec) for 1 h. MC38 cells were preincubated with the indicated concentrations of NanoAlb‐proDOX for 8 h and then activated CD8^+^ T cells and 45 μg/ml anti‐PD‐L1 antibody were added for another 18 h of coculture. Proliferation of the CD8^+^ T cells was determined using flow cytometry by measuring CFSE fluorescence.

### 5.12 Histology analysis

After the mice were sacrificed, the major organs were recovered from the necropsy and fixed with 10% neutral buffered formalin. Afterward, the organs were embedded in paraffin and sectioned at 5 mm, and hematoxylin and eosin (H&E) staining was performed for histological examination.

### 5.13 IHC analysis

After the mice were sacrificed, the tumor tissues were harvested and fixed with 4% paraformaldehyde. Formalin‐fixed, paraffin‐embedded samples were cut into 4 μm sections. IHC was performed on the Leica Bond automated staining platform. The CD8 antibody (CST, #98941) was run at 1:400 dilution using the Leica Biosystems Refine Detection Kit with EDTA antigen retrieval. Images were captured using a Zeiss LSM600 microscope. Five randomly selected square areas (1  mm^2^ each) in the tumor were evaluated. The average total number of positive cells and the integral optical density in the five areas were analyzed by ImageJ. The percentage of CD8‐positive cells with respect the total cells in tumors was subjected to GraphPad Prism. Three representative views were counted for each group.

### 5.14 Statistical Analyses

All the values are presented as the mean ± S.E.M. The number n represents technical replicates or the number of mice used. All statistical analyses were performed with GraphPad Prism 7 software. Significance tests between two groups were performed using two‐tailed unpaired *t* tests. Significance was defined as *p* > 0.05 n.s. not significant, *p < 0.05, **p < 0.01, ***p < 0.001, ****p < 0.0001.

## AUTHOR CONTRIBUTIONS

**Long Chen:** Conceptualization (equal); data curation (equal); formal analysis (equal); investigation (equal); methodology (lead); visualization (lead); writing – original draft (lead); writing – review and editing (lead). **Nuo Xu:** Conceptualization (equal); data curation (equal); formal analysis (equal); investigation (equal); methodology (lead); visualization (lead); writing – original draft (lead); writing – review and editing (lead). **Pan Wang:** Conceptualization (equal); data curation (equal); formal analysis (equal); investigation (equal); methodology (lead); visualization (lead); writing – review and editing (equal). **Haichuan Zhu:** Formal analysis (supporting); investigation (supporting); methodology (supporting); visualization (supporting); writing – original draft (supporting). **Zijian Zhang:** Formal analysis (supporting); investigation (supporting); methodology (supporting); visualization (supporting). **Zhanqun Yang:** Investigation (supporting); methodology (supporting); visualization (supporting). **Wenyuan Zhang:** Investigation (supporting); methodology (supporting); visualization (supporting). **Jian Lin:** Conceptualization (equal); data curation (equal); formal analysis (equal); funding acquisition (lead); methodology (lead); project administration (lead); supervision (lead); visualization (equal); writing – original draft (lead); writing – review and editing (lead). **Hongyan Guo:** Conceptualization (equal); data curation (equal); formal analysis (equal); funding acquisition (lead); methodology (lead); project administration (lead); supervision (lead); visualization (equal); writing – original draft (lead); writing – review and editing (lead).

## CONFLICT OF INTERESTS

The authors declare no competing financial interest.

## Supporting information


**Appendix S1** Supporting Information

## Data Availability

Data available on request from the corresponding authors.
