# Supplementary material for: Nanoalbumin–prodrug conjugates prepared via a thiolation‐and‐conjugation method improve cancer chemotherapy and immune checkpoint blockade therapy by promoting CD8 + T‐cell infiltration
Source: Bioeng Transl Med. 2022 Jul 30;8(1):e10377. doi: 10.1002/btm2.10377 (PMC9842047; doi:10.1002/btm2.10377)
Supplement: Supplementary file 1 — Appendix S1 Supporting Information [file BTM2-8-e10377-s001.docx]

**Supporting Information**

**NanoAlbumin-Prodrug Conjugates Prepared via a Thiolation-and-Conjugation Method Improve Cancer Chemotherapy and Immune Checkpoint Blockade Therapy by Promoting CD8^+^ T Cell Infiltration**

Long Chen ^a,1^, Nuo Xu ^a,1^, Pan Wang ^b,1^, Haichuan Zhu ^c^, Zijian Zhang ^c^, Zhanqun Yang ^a^, Wenyuan Zhang ^a^, Hongyan Guo ^b,^*, Jian Lin ^a,^*

^a^ Department of Pharmacy, Peking University Third Hospital, College of Chemistry and Molecular Engineering, Peking University, Beijing 100191, China

^b^ Department of Obstetrics and Gynecology, Peking University Third Hospital, Beijing 100191, China

^c^ Institute of Biology and Medicine, College of Life and Health Sciences, Wuhan University of Science and Technology, Wuhan 430081, China

^1^ These authors contribute equally to this work

* Corresponding Author：

Hongyan Guo, Email: bysyghy@163.com

Jian Lin, Email: linjian@pku.edu.cn


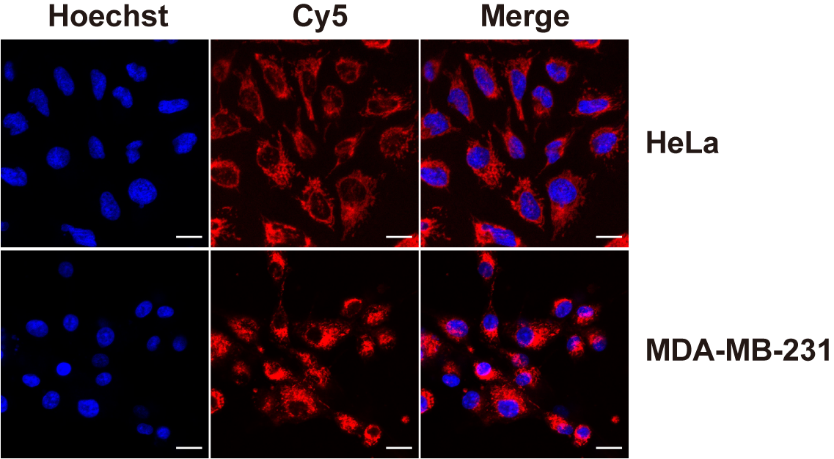


**Figure S1.** Confocal images showing the uptake of albumin by cancer cells. Human serum albumin was covalent labeled with fluorescent dye Cy5 (HSA-Sulfo-Cy5). 10μM HSA-Sulfo-Cy5 was incubated with cancer cells and images were taken 24 fours later with Zeiss LSM 700 confocal microscope. Scale bar: 20 μm.


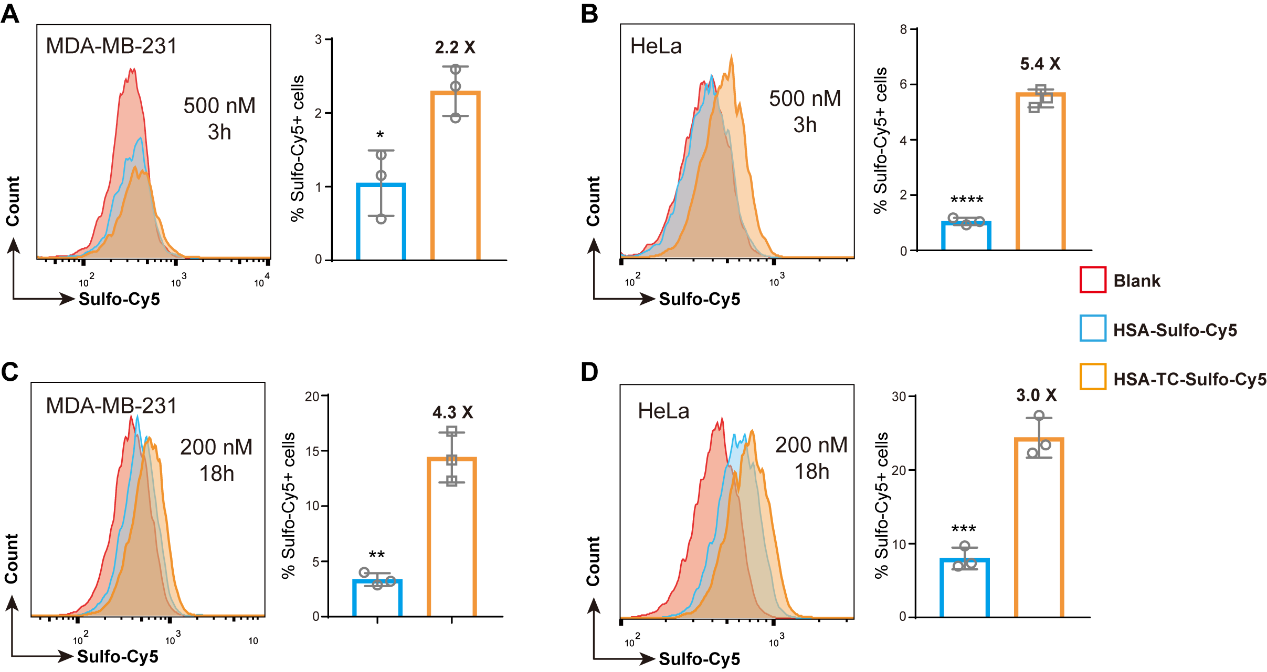


**Figure S2.** Flow cytometry showing the uptake of HSA-TC-Sulfo-Cy5 by two cancer cell lines. (A) Flow cytometry showing the uptake of HSA-TC-Sulfo-Cy5 by MDA-MB-231 cancer cells incubated for 3h at the concentration of 500 nM. (B) Flow cytometry showing the uptake of HSA-TC-Sulfo-Cy5 by HeLa cancer cells incubated for 3h at the concentration of 500 nM. (C) Flow cytometry showing the uptake of HSA-TC-Sulfo-Cy5 by MDA-MB-231 cancer cells incubated for 18h at the concentration of 200 nM. (D) Flow cytometry showing the uptake of HSA-TC-Sulfo-Cy5 by HeLa cancer cells incubated for 18h at the concentration of 200 nM. Percentages of the Sulfo-Cy5 positive cells were gated and fold changes between two conjugates were marked. Data are presented as the mean±SEM. n=3 technical replicates. ^*^ p < 0.05, ^**^ p < 0.01, ^***^ p < 0.001, ^****^ p < 0.0001.  ^*^ p < 0.05, ^**^ p < 0.01, ^***^ p < 0.001, ^****^ p < 0.0001.


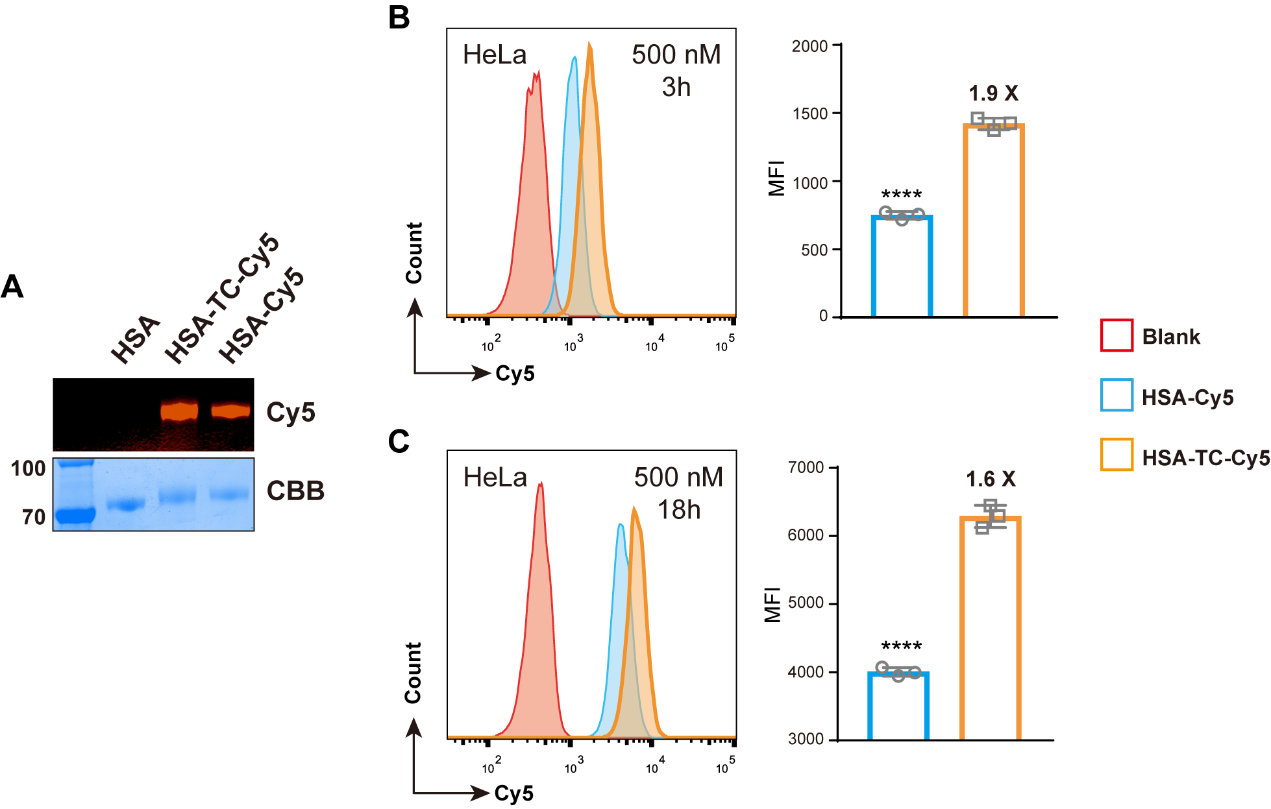


**Figure S3.** (A) SDS-PAGE analysis of HSA-TC-Cy5. Up: Cy5 fluorescence. Down: Commassie blue stating. (B) Flow cytometry showing the uptake of HSA-TC-Cy5 by HeLa cancer cells incubated for 3h at the concentration of 500 nM. (C) Flow cytometry showing the uptake of HSA-TC-Cy5 by HeLa cancer cells incubated for 18h at the concentration of 500 nM. Mean fluorescence intensity was plotted and fold changes between two conjugates were marked. Data are presented as the mean±SEM. n=3 technical replicates. ^****^ p < 0.0001.


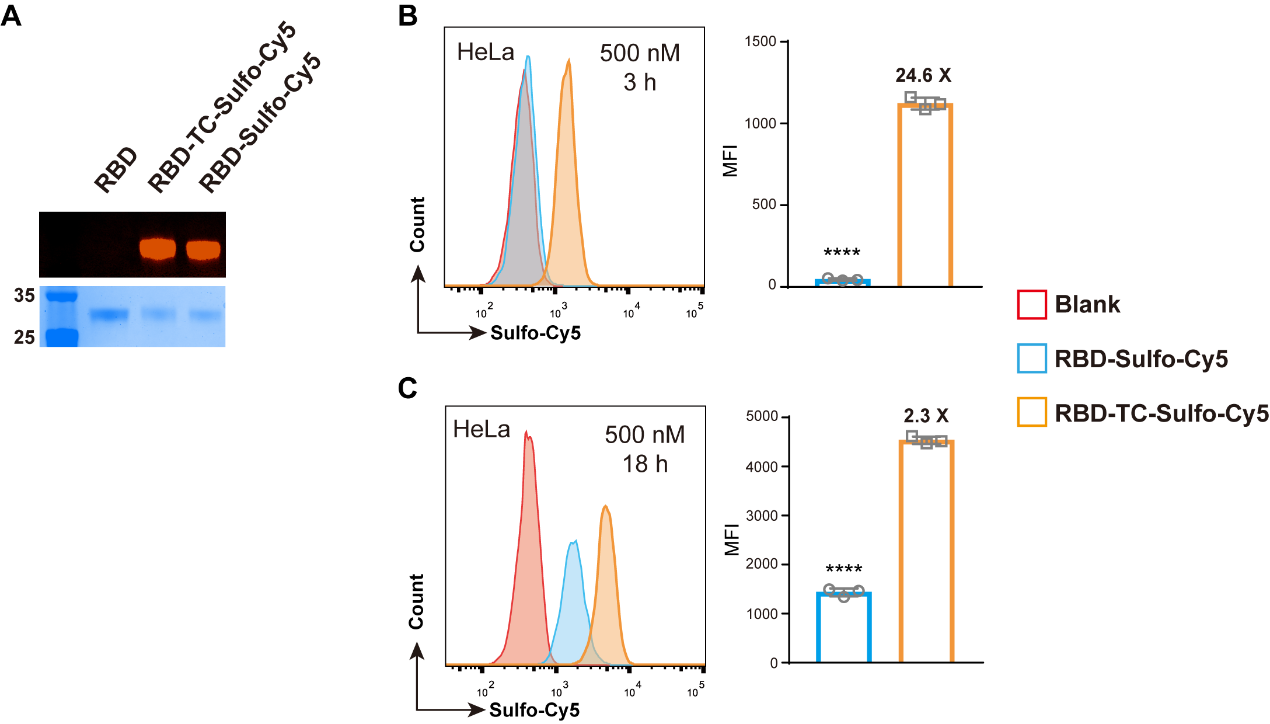


**Figure S4.** (A) SDS-PAGE analysis of RBD-TC-Sulfo-Cy5. Fluorescent bands indicated that RBD was successfully labeled with Sulfo-Cy5 using two different methods. Up: Cy5 fluorescence. Down: Commassie blue stating. (B) Flow cytometry showing the uptake of RBD-TC-Sulfo-Cy5 by Hela cancer cell incubated for 3h at the concentration of 500 nM. (C) Flow cytometry showing the uptake of RBD-TC-Cy5 by Hela cancer cell incubated for 18h at the concentration of 500 nM. Mean fluorescence intensity was plotted and fold changes between two conjugates were marked. Data are presented as the mean±SEM. n=3 technical replicates. ^****^ p < 0.0001.


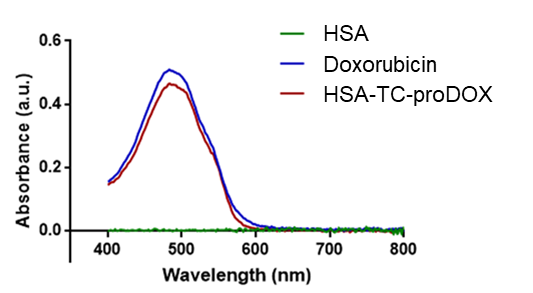


**Figure S5.** UV-Vis absorbance of HSA, Doxorubicin and HSA-TC-proDOX


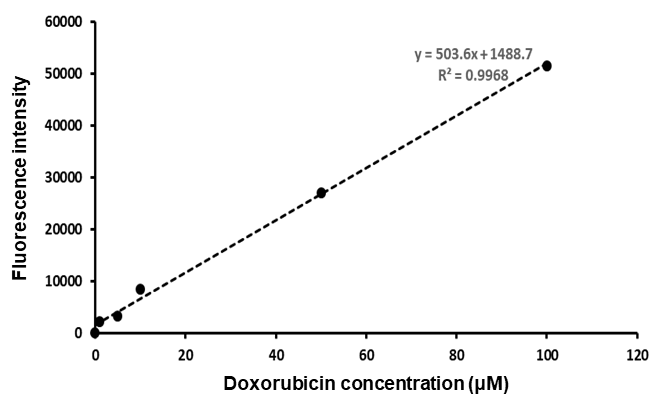


**Figure S6.** Standard curve of doxorubicin plotted by doxorubicin fluorescence with excitation at 485 nm and emission at 590 nm.

**Table 1** Determine of the conjugation efficacy of doxorubicin on HSA

|  | Protein concentration | Fluorescence of  doxorubicin | Doxorubicin concentration | Average doxorubicin No. per HSA |
| --- | --- | --- | --- | --- |
| HSA-TC-proDOX( NanoAlb-proDOX) | 1.46 mg/mL | 34923 | 66.4 μM | ~ 3.0 |


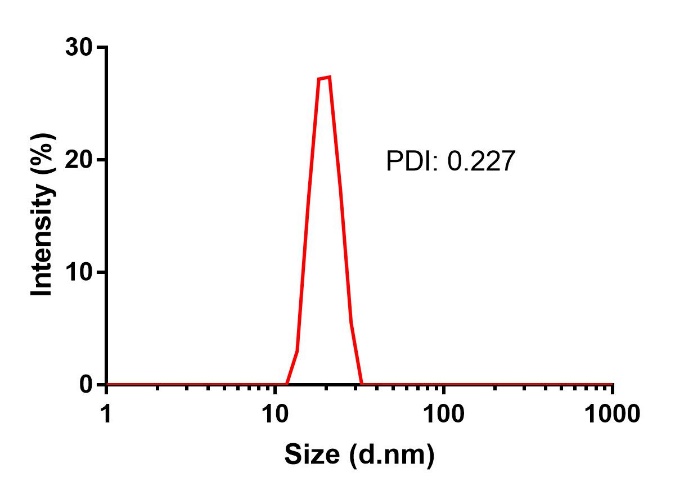


**Figure S7.** Dynamic light scattering analysis of the HSA-TC-proDOX. The measured particle size was 21nm.


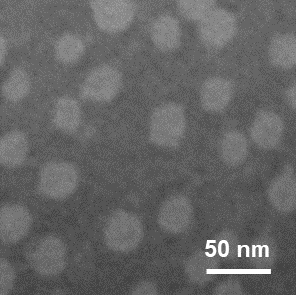


**Figure S8.** TEM image of HSA-TC-Sulfo-Cy5


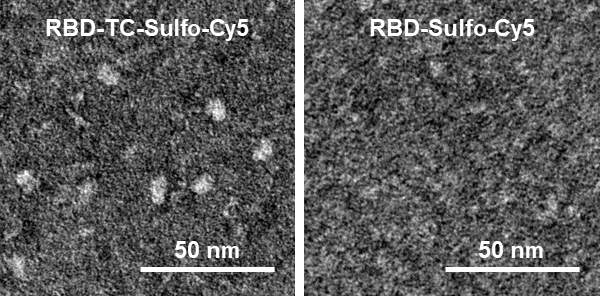


**Figure S9.** TEM image of RBD-TC-Sulfo-Cy5 and RBD-Sulfo-Cy5


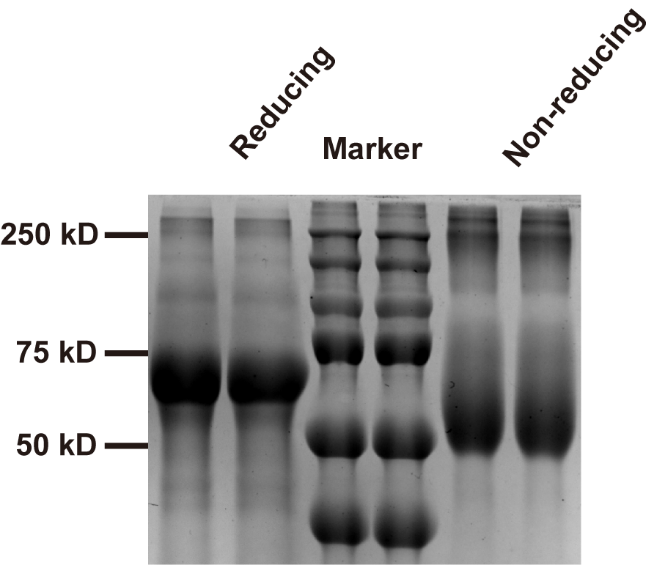


**Figure S10.** Reducing and non-reducing SDS-PAGE analysis of HSA-TC-proDOX. In non-reducing condition high molecular bands was observed and disappeared in the reducing condition indicating the presence of intermolecular disulfide bonds.


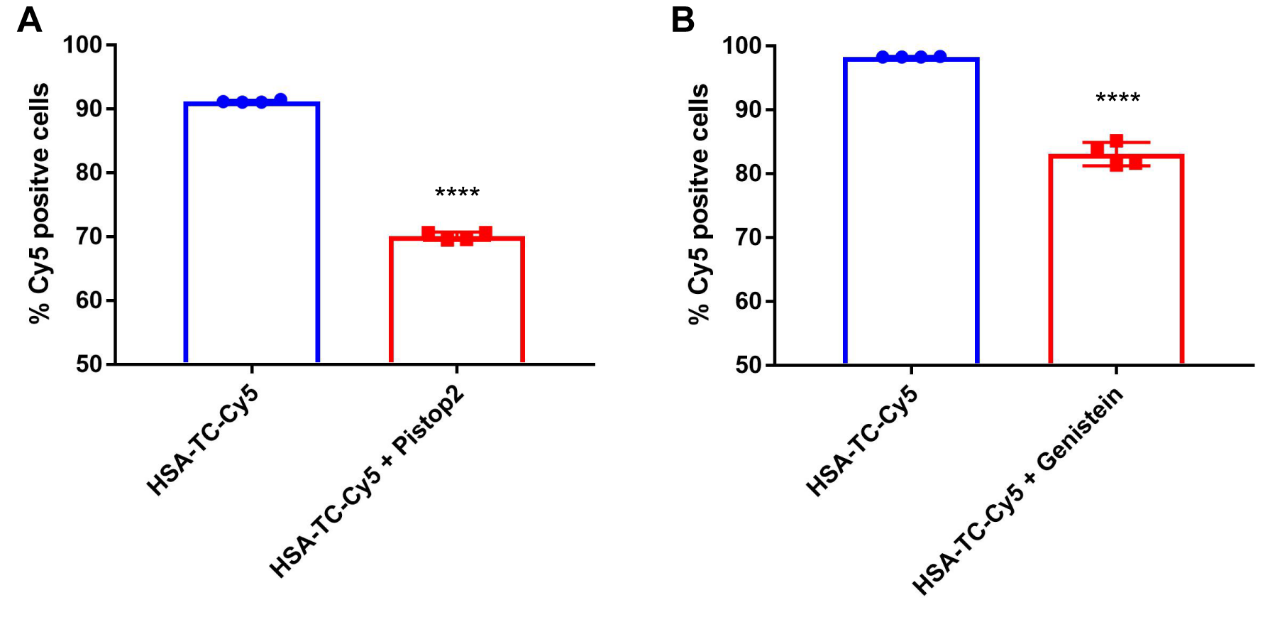


**Figure S11.** Reduced endocytosis of HSA-TC-Cy5 by Pistop 2 (A) and Genistein (B) in HeLa cells. HeLa cells were first treated with Pistop 2 (5 μM) or Genistein (100 μM) one hours before the addition of 200 nM HSA-TC-Cy5. After another one-hour incubation at 37 ℃, cells were resuspended in PBS and subjected to flow cytometry. Cy5 positive cells were gated. Data are presented as the mean±SEM. N=4 technical replicates. ^**^ p < 0.0001.


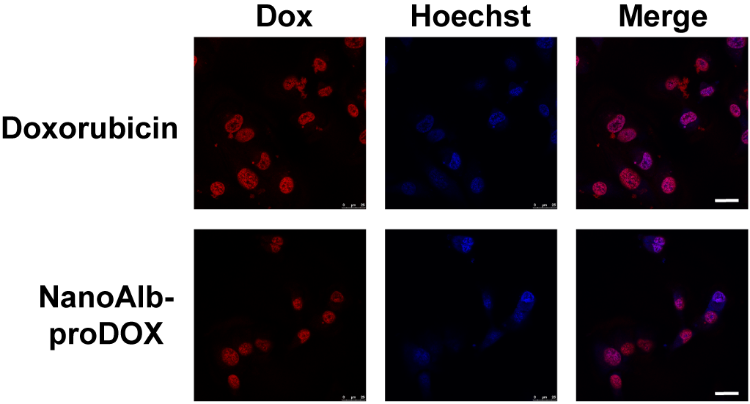


**Figure S12.** Confocal images showing the nucleus localization of doxorubicin and NanoAlb-proDOX. Nucleus was stained with Hoechst 33342. Scale bar: 25 μm.


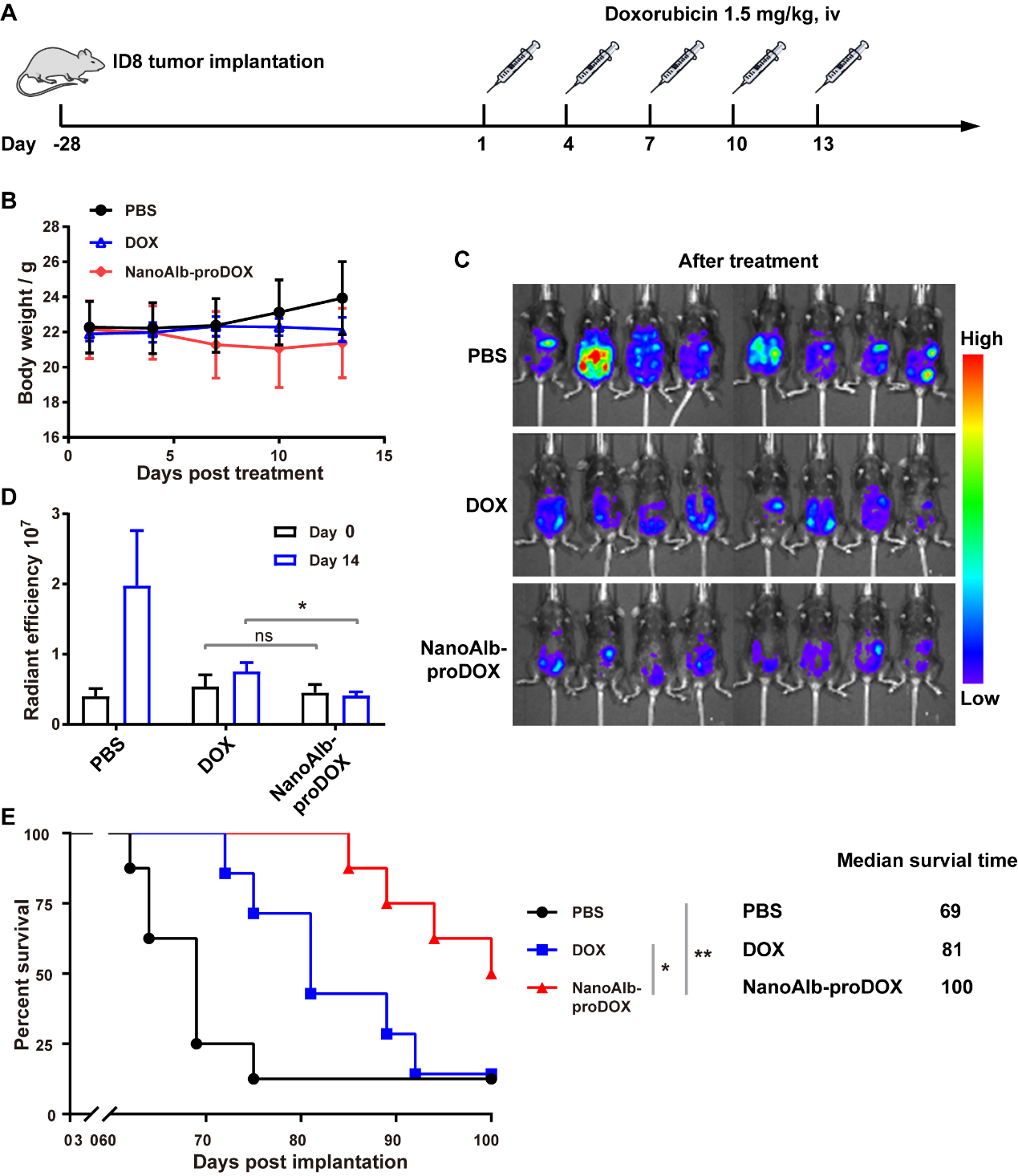


**Figure S13.** Antitumor activity of NanoAlb-proDOX in ascites xenograft model of ovarian cancer. (A) Schematic representation of the design of animal experiments. (B) Body weight curves of the treated mice. (C) Representative bioluminescence images of the mice post treatment on day 14. Mice bearing ID8-Luc ascites xenograft model were injected with 1mg/mL luciferin (100 μL) and 10 minutes later, the mice were subjected to live animal bioluminescence imaging. Low bioluminescence was observed for NanoAlb-proDOX treated mice, indicating a good anti-ascites activity of NanoAlb-proDOX. (D) Quantitative analysis of the bioluminescence imaging before and after treatment. (E) Survival curves of mice treated with different drugs. NanoAlb-proDOX treatment greatly enhanced the survival of the mice. Data were presented as mean±SEM. n=8 mice. ^*^ p < 0.05, ^**^ p < 0.01.


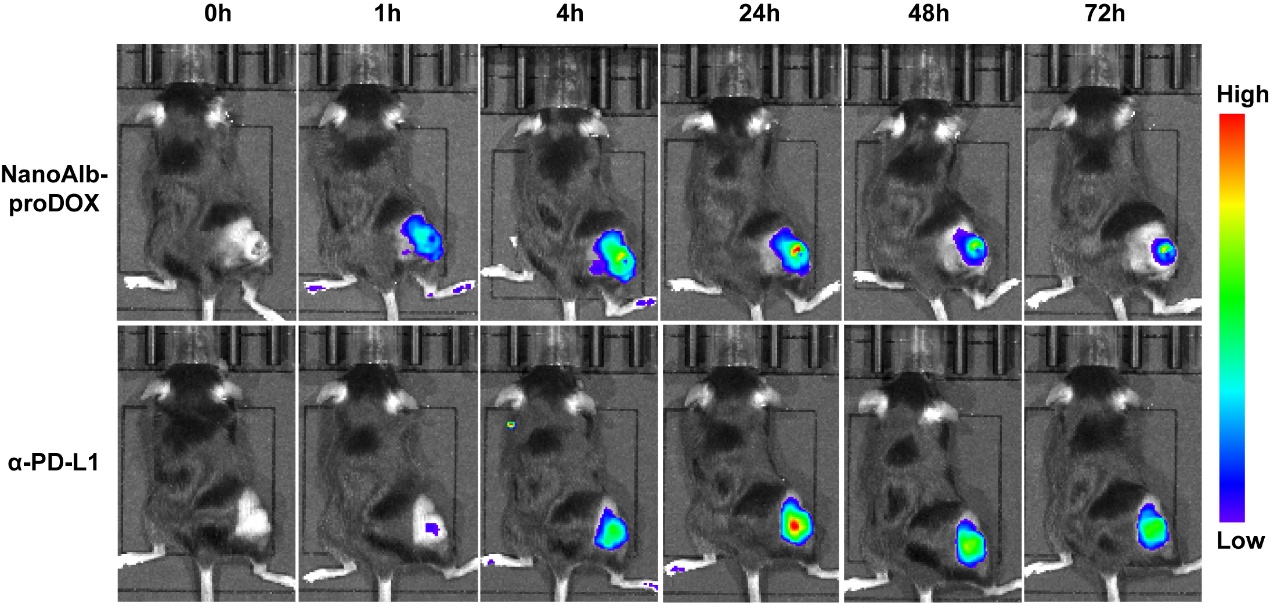


**Figure S14.** Accumulation of NanoAlb-proDOX and anti-PD-L1 antibody in MC38 tumor. Cy5 labeled HSA-TC-proDOX or α-PD-L1was intravenously injected to mice bearing MC38 subcutaneous tumor and fluorescence imaging was performed at different timepoints using living animal imaging. Accumulation of HSA-TC-proDOX and α-PD-L1 in tumor regions was observed.


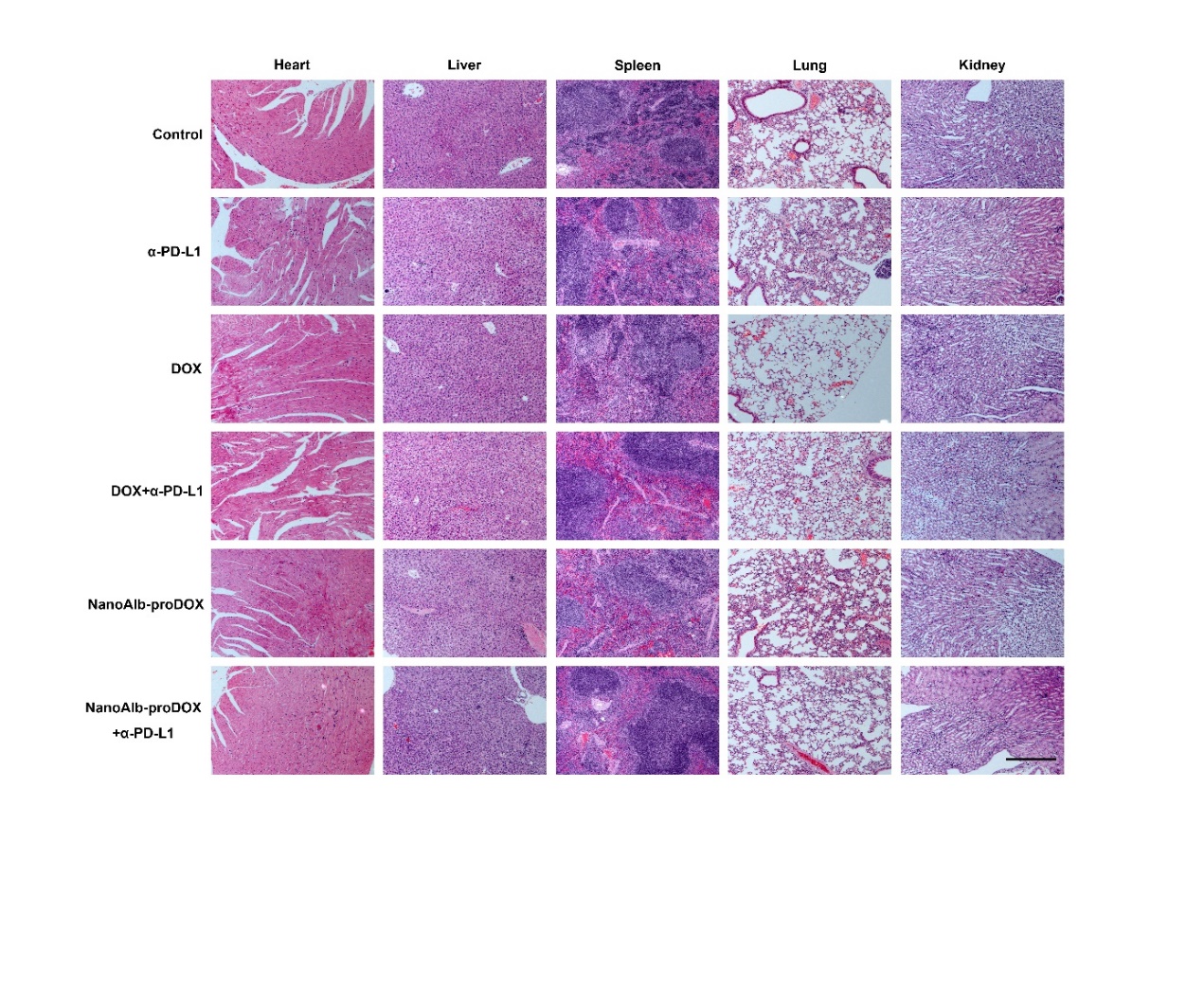


**Figure S15.** H&E staining images of major organs collected from experiment performed in Figure 5. No morphological changes were observed for organs from drug treated mice compared to control mice. Scale bar: 50 μm.


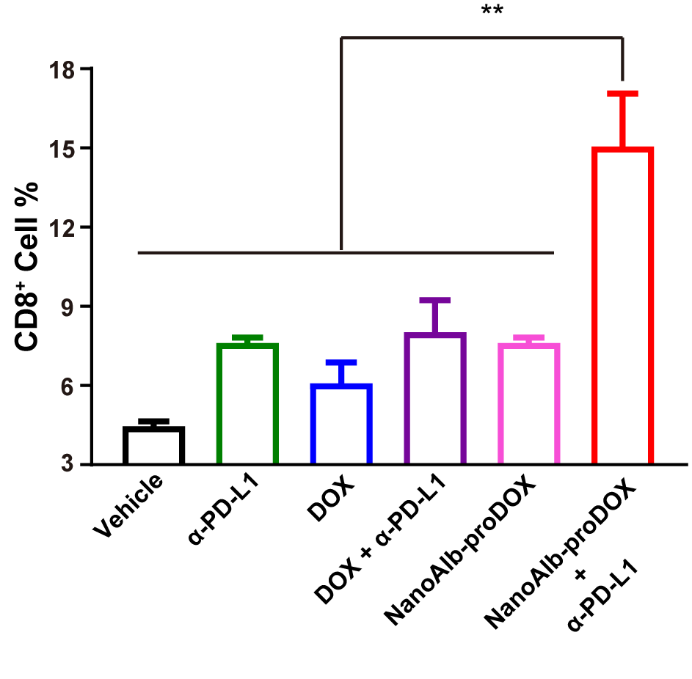


**Figure S16.** Quantitative analysis of CD8^+^ T cells from IHC images shown in Figure 6. IHC images showing in figure 6 were quantitatively analyzed using Image J software by counting the percentage of CD8 positive cells versus the whole cells in one field view. Three representative views were counted for each group. Data are presented as mean±SEM. n=3. n represented that tissues from 3 mice in each group were measured. ^**^ p < 0.01.


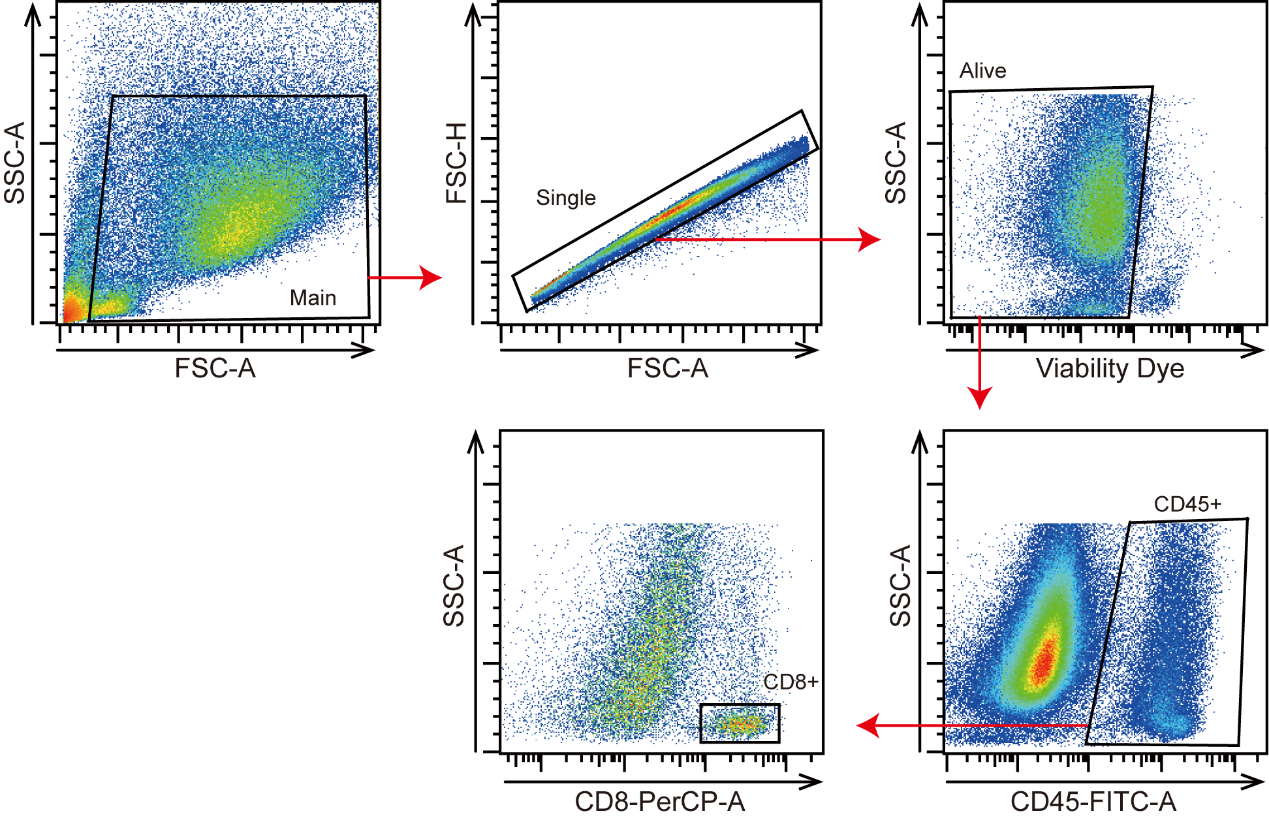


**Figure S17.** Representative flow cytometry images for the gating of CD8^+^ cells from tumor infiltrated CD45^+^ immunocytes. Single and live cells were analyzed in each experiment.


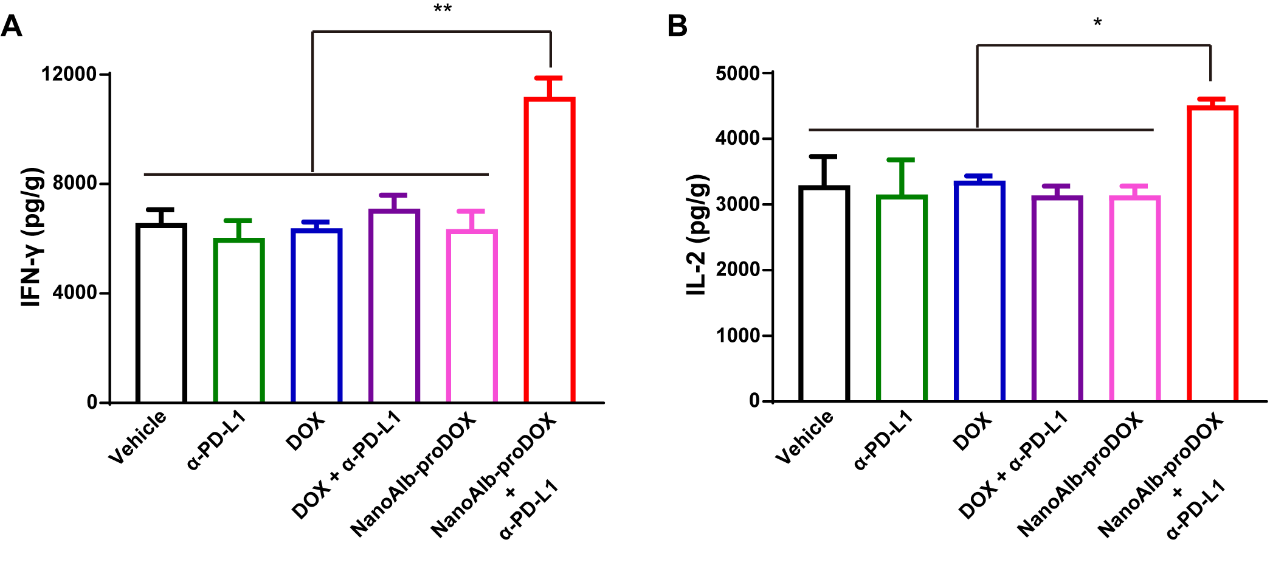


**Figure S18.** Concentrations of IFN-γ (A) and IL-2 (B) in tumor tissues measured by ELISA. Three individual tumor tissues from each group were acquired and homogenized in cold PBS. The concentration of the cytokines in the supernatant were measured by ELISA. Data were presented as mean±SEM. n=3. n represented that tissues from 3 mice in each group were measured. ^*^ p < 0.05, ^**^ p < 0.01.
